# A novel pray optimization algorithm for six degree of freedom robotic arm trajectory planning

**DOI:** 10.1038/s41598-026-63211-w

**Published:** 2026-07-28

**Authors:** Assem F. Alabu-Husain, Mostafa A. ElBahloul, Mahmoud M. Saafan, Eman M. El-Gendy

**Affiliations:** 1 Mechatronics Engineering Department, Faculty of Engineering, Mansoura University, Mansoura, Egypt; 2 Production Engineering and Mechanical Design Department, Faculty of Engineering, Mansoura University, Mansoura, Egypt; 3 Computers and Control Systems Engineering Department, Faculty of Engineering, Mansoura University, Mansoura, Egypt; 4 Faculty of Engineering, Mansoura National University, Gamasa, Egypt

**Keywords:** Metaheuristic optimization, Pray optimization algorithm, Benchmark functions, Robotic arm, Trajectory planning, Engineering, Mathematics and computing

## Abstract

This study introduces the Pray Optimization Algorithm (POA), a novel metaheuristic inspired by the procedural rituals of Islamic pray, designed to solve complex engineering and robotic manipulator problems. The mathematical model is structured into three distinct phases: Phase I simulates searching for a suitable mosque; Phase II models congregational alignment (lining up for pray); and Phase III implements a cumulative scoring system to drive convergence. The efficacy of the POA is initially validated on the CEC2017 benchmark suite through comparative analysis with six high-performing metaheuristic algorithms. The algorithm’s statistical superiority is subsequently confirmed using the Wilcoxon rank-sum and Friedman tests. To verify its practical applicability, the POA is deployed to solve three classical real-world engineering optimization problems. Ultimately, the algorithm demonstrates superior performance when compared against five state-of-the-art methods in optimizing the trajectory planning of a 6-DOF industrial robotic arm. These findings substantiate the effectiveness of the proposed POA in navigating constrained, real-world engineering search spaces.

## Introduction

Optimization generally entails seeking an objective function’s extremum under constraints, the landscape of modern engineering has evolved. Contemporary applications now demand solutions to problems characterized by vast search spaces and high-dimensional objectives, reflecting an increase in both complexity and scale^1^. As flexible techniques independent of gradient information, metaheuristic algorithms excel in global search and robustness when addressing black-box problems. Their capacity to operate using only objective function evaluations renders them highly effective for practical implementations in engineering and machine learning^2^.

In recent years, numerous metaheuristic algorithms have been proposed to solve optimization problems. However, according to the principle of No Free Lunch theorem^3^, no single algorithm can solve all optimization problems. Therefore, many researchers have developed various algorithms attempting to balance between exploration and exploitation to avoid trapped at the local optima and reach to the global optimum with more accuracy^4^.

**Fig. 1:**
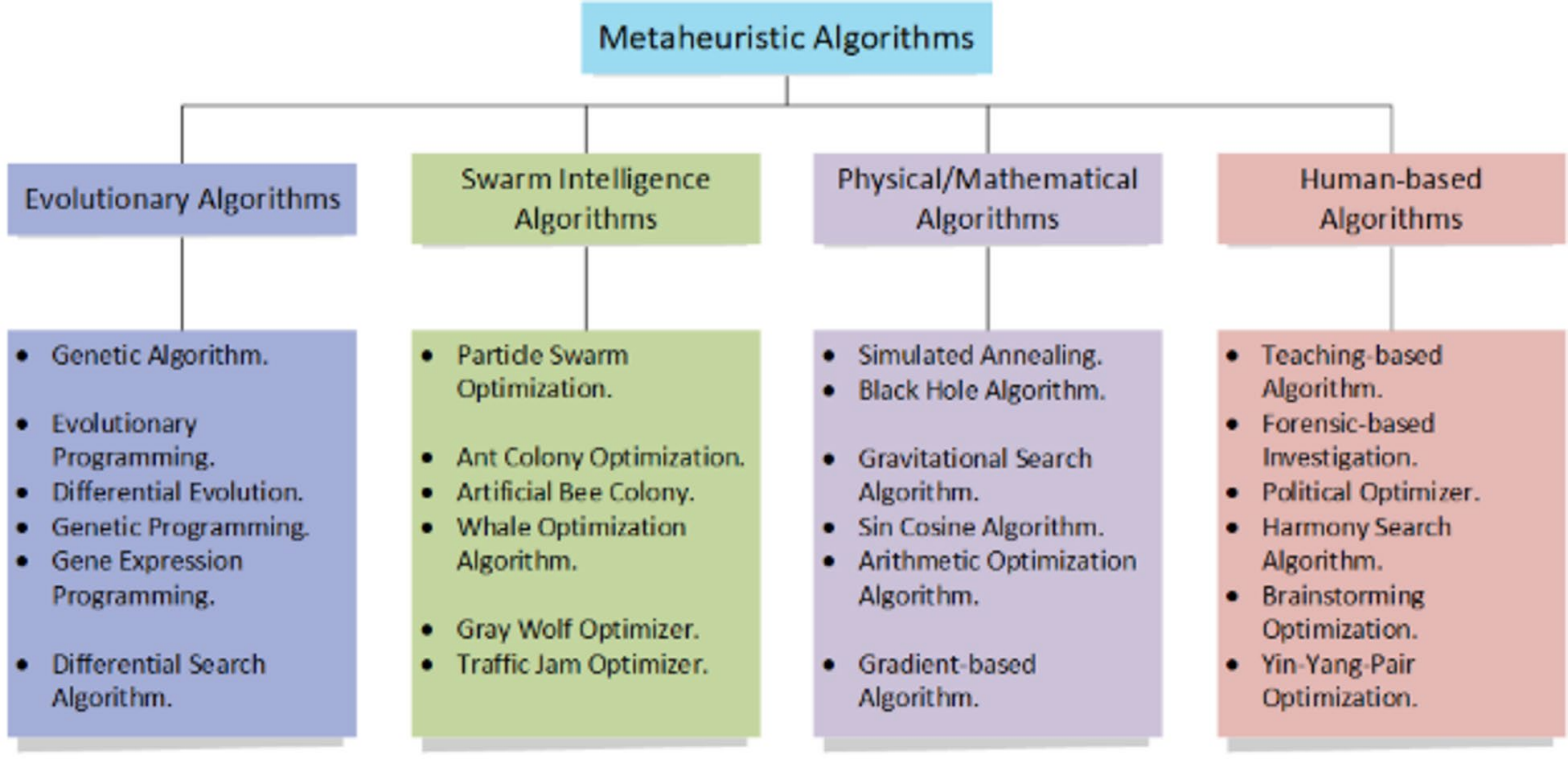
Classification of metaheuristic algorithms.

This section provides a comprehensive review of metaheuristic algorithms. Based on their underlying inspiration, these algorithms are generally classified into four main categories: swarm intelligence, evolutionary algorithms, physics/mathematics-based algorithms, and human-inspired algorithms, as shown in Fig. 1.

Evolutionary Algorithms (EAs) mimic the mechanics of biological evolution, utilizing genetic operators such as selection, crossover, mutation, and elimination to evolve candidate solutions. For instance, The Genetic Algorithm (GA)^5^ mimics natural selection using crossover and mutation to propagate superior individuals and converge on the global optimum. Other evolutionary algorithms are introduced for solving optimization algorithms include evolutionary programming^6^, genetic programming^7^, water flow optimizer (WFO)^8^, etc.

Swarm Intelligence constitutes a distinct class of metaheuristics inspired by the collective, self-organized behaviors of natural systems. These algorithms emulate the interactions of decentralized agents, such as bird flocks or insect colonies. One of the most popular swarm intelligence algorithms is Particle Swarm Optimization PSO^9^, This algorithm draws inspiration from the foraging behavior of bird flocks. By facilitating information exchange among individuals during stochastic searches, the algorithm enables the population to collectively converge toward the global optimum. The Ant Colony Optimization (ACO)^10^ mimics the path-finding strategies of ants, where individuals are guided by the concentration of pheromones deposited by preceding colony members. Another popular algorithm is gray wolf optimizer (GWO)^11^, The algorithm mimics the leadership structure where the alpha wolf guides the pack and mathematically models specific predatory strategies, such as encircling and attacking the prey. It is noteworthy that most metaheuristic algorithms are classified as swarm intelligence, Prominent examples of such algorithms, artificial bee colony (ABC)^12^, Harris Hawks optimization (HHO)^13^, whale optimization algorithm (WOA)^14^.

The category of physics-based metaheuristics mimics the behavior of physical systems. In these algorithms, the search process is driven by the simulation of physical rules that dictate the interaction and state changes of the agents. For instance, simulated annealing (SA)^15^ draws inspiration from the annealing of solids. By simulating the gradual decrease in temperature, the algorithm regulates the search behavior: distinct, random fluctuations at high temperatures facilitate global exploration, while lower temperatures restrict movement to fine-tune the solution toward a stable energy minimum.

Recent literature highlights the effectiveness of algorithms inspired by mathematical systems. A notable example is the Sine Cosine Algorithm (SCA)^16^, which employs trigonometric principles to balance exploration and exploitation.

The final category, human-inspired algorithms, draws its theoretical framework from the complex social, political, and cognitive interactions observed in human societies. For instance, the forensic-based investigation algorithm (FBI)^17^, political optimizer (PO)^18^, and Teaching-Learning-Based Optimization (TLBO)^19^ models the influence of a teacher on learners. This classroom-inspired approach is widely recognized for its strong performance and convergence speed in diverse engineering applications.

However, a comprehensive review of the literature reveals that to date, no algorithm has been developed that is inspired by the process of Islamic pray, so a novel metaheuristic algorithm named pray optimization algorithm (POA) is proposed in this paper.

POA mimics the procedure of the pray in Islam, through just three phases, moving to the mosque, lining up for the pray, and the cumulative score phase. In the moving to the mosque phase, individuals search freely for the suitable mosque to explore the search space at the beginning then exploit to the global optimum at the end, In the second phase, candidate solutions begin to line up in rows after the Imam, and in the cumulative phase, each candidate tries to reach the highest score where as individual pray more as he has higher score to find the global best and accelerate convergence.

Trajectory planning remains a fundamental challenge in applied robotics research. It involves the precise calculation of a continuous motion profile—dictating the intermediate waypoints, velocity, acceleration, and orientation—required to transition the end-effector from an initial configuration to a target pose. The primary objective of this process is to ensure the manipulator navigates its workspace in a smooth, dynamically controlled, and vibration-free manner, while strictly avoiding any environmental collisions^20^. In practice, robotic manipulators rarely execute direct, linear transitions from an initial configuration to a final target. This limitation arises from joint-space non-linearities, differential actuator velocities, and the necessity for environmental obstacle avoidance. Consequently, it is essential to generate a structured trajectory that routes the end-effector through carefully calculated intermediate waypoints. Determining these waypoints requires the precise synchronization of velocity, acceleration, and distance parameters across all joint axes a kinematic challenge that scales exponentially in complexity with the robot’s degrees of freedom.

The primary objective of this research is to achieve time-optimal trajectory planning for 6-DOF industrial robotic manipulators. To realize this goal, a novel metaheuristic algorithm, termed the Pray Optimization Algorithm (POA), is introduced. This study specifically focuses on minimizing the total motion execution time while strictly adhering to established kinematic constraints to ensure the mechanical feasibility of the generated trajectories. Ultimately, by resolving these high-dimensional optimization challenges, this research seeks to significantly enhance the operational efficiency and productivity of advanced robotic systems in industrial applications.

The main contributions of this work as follows:

1. A novel metaheuristic algorithm called Pray Optimization Algorithm is proposed, which is inspired by three main stages of Islamic pray process, the fundamental of pray as a swarm-based, and spiritual human behavior.
2. The optimization performance of the proposed POA is evaluated using selected functions from the CEC2017 benchmark suite^21^. Furthermore, a comparative analysis is conducted against six well-established algorithms to verify the proposed algorithm superiority and competitiveness.
3. The proposed algorithm’s performance is evaluated on three practical engineering case studies. These results are contrasted with leading algorithms to confirm POA’s potential in solving actual industrial problems.
4. Finally, the practical utility and superiority of the proposed POA are demonstrated through its application to a critical challenge in robotics: optimizing the trajectory planning for a 6-DOF industrial robotic arm. These high-dimensional real-world applications serve to validate the algorithm’s effectiveness in generating precise and efficient motion profiles for advanced robotic systems.

The remainder of this paper is organized as follows: Sect.  2 presents a detailed review of related work. Section  3 delineates the proposed algorithm, detailing its inspiration, mathematical formulation, and implementation pseudocode. Section  4 presents the experimental results and analysis based on benchmark functions. Section  5 demonstrates the method’s practical applicability by solving three complex engineering design problems, Sect.  6 establishes the practical utility and robustness of the POA through the resolution of time-optimal trajectory planning of 6-DOF robotic arm. Finally, Sect.  7 summarizes the conclusions and directions for future work.

## Related work

Contemporary approaches to robotic arm trajectory planning generally fall into two broad categories: traditional deterministic methods and swarm intelligence-based optimization algorithms. Recently, swarm intelligence has gained significant traction for its ability to navigate complex, non-linear constraints. Demonstrating this, Han et al.^22^ employed an enhanced Sparrow Search Algorithm (SSA) to address the time-optimal trajectory planning problem for a 6-DOF robotic manipulator. By utilizing a 3-5-3 piecewise polynomial interpolation within the joint space, their proposed optimization framework successfully achieved an approximate 31% reduction in total motion execution time. Similarly, Ekrem et al.^20^ utilized Particle Swarm Optimization (PSO) coupled with quintic polynomial interpolation to generate time-optimized, vibration-free joint trajectories for a 6-DOF robotic manipulator. Targeting the optimization challenges of robotic manipulator trajectories, Leng et al.^23^ introduced the Enhanced Crayfish Optimization Algorithm (ECOA). By modifying the standard COA with chaotic population initialization and opposition-based learning strategies, the authors successfully enhanced the algorithm’s search efficiency. In applied trajectory planning experiments, the ECOA yielded highly stable convergence profiles and outperformed standard baselines, reducing overall trajectory execution costs by 15% relative to the best competing metaheuristic. Targeting time-optimal motion generation, Gu et al.^24^ successfully applied an improved Harris Hawks Optimization (HHO) algorithm to the time-optimal trajectory planning of a UR5 manipulator. By modifying the algorithm’s exploration stage and utilizing a 3-5-3 piecewise polynomial interpolation, the authors ensured continuous joint-space motion while significantly reducing both convergence metrics and total trajectory execution time. Expanding metaheuristic applications to cooperative dual-arm systems, Peñacoba-Yagüe et al.^25^ proposed a feasibility-first optimization methodology for time-efficient, collision-free payload transport. Through comparative benchmarking, their study established Particle Swarm Optimization (PSO) as the most effective algorithm—significantly outperforming WOA and GOA—for balancing rapid collision avoidance with optimal trajectory execution times. To balance time efficiency and energy consumption in complex industrial workspaces, Elgohry et al.^26^ developed a hybrid Whale Genetic Algorithm (WGA) tailored for 6-DOF robotic trajectory planning. By combining the distinct search strengths of WOA and GA, their methodology successfully generated feasible motion profiles that reduced total execution time by 44% and energy consumption by (5%-10%), with theoreticl oucomes rigorously validated on a physical KUKA manipulator.

## Pray optimization algorithm

### Inspiration

The main idea of the algorithm is mimicking the process of Islamic pray in some parts, where the prayers (individuals) try to reach high score by going to the suitable mosque and by praying at the first line after Imam.

#### Phase I: searching and moving to the suitable mosque

The algorithm initializes the number of mosques based on the dimensionality of the search space. For problems with fewer than 10 decision variables, the population size is set equal to the number of variables. Conversely, for high-dimensional problems (10 or more variables), the population size is scaled to one-tenth of the variable count.

**Fig. 2:**
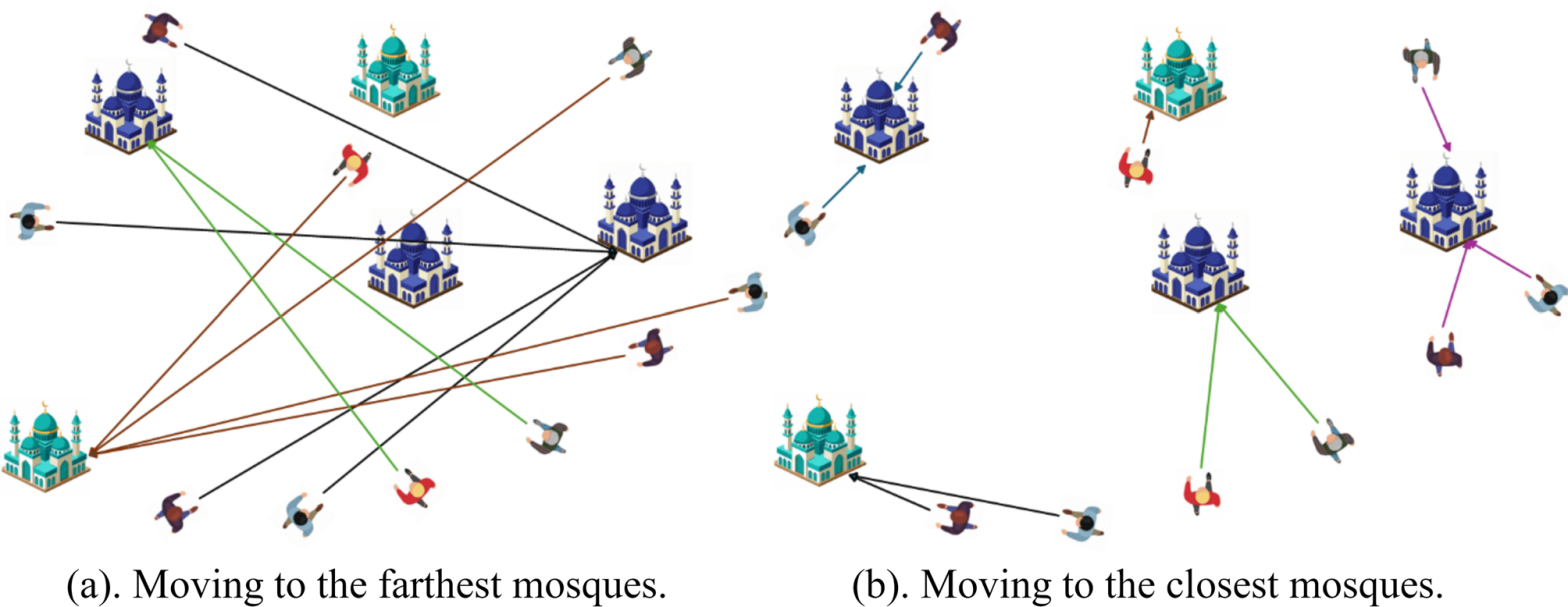
Individuals moving strategies.

At the beginning of iterations as shown in Fig. 2, the prayers search for the farthest mosque to reach high score (more exploration), otherwise at the latest iterations the individuals are moving to the closest mosque because they do not want to miss the score of the pray.

#### Phase II: lining up for the pray

**Fig. 3:**
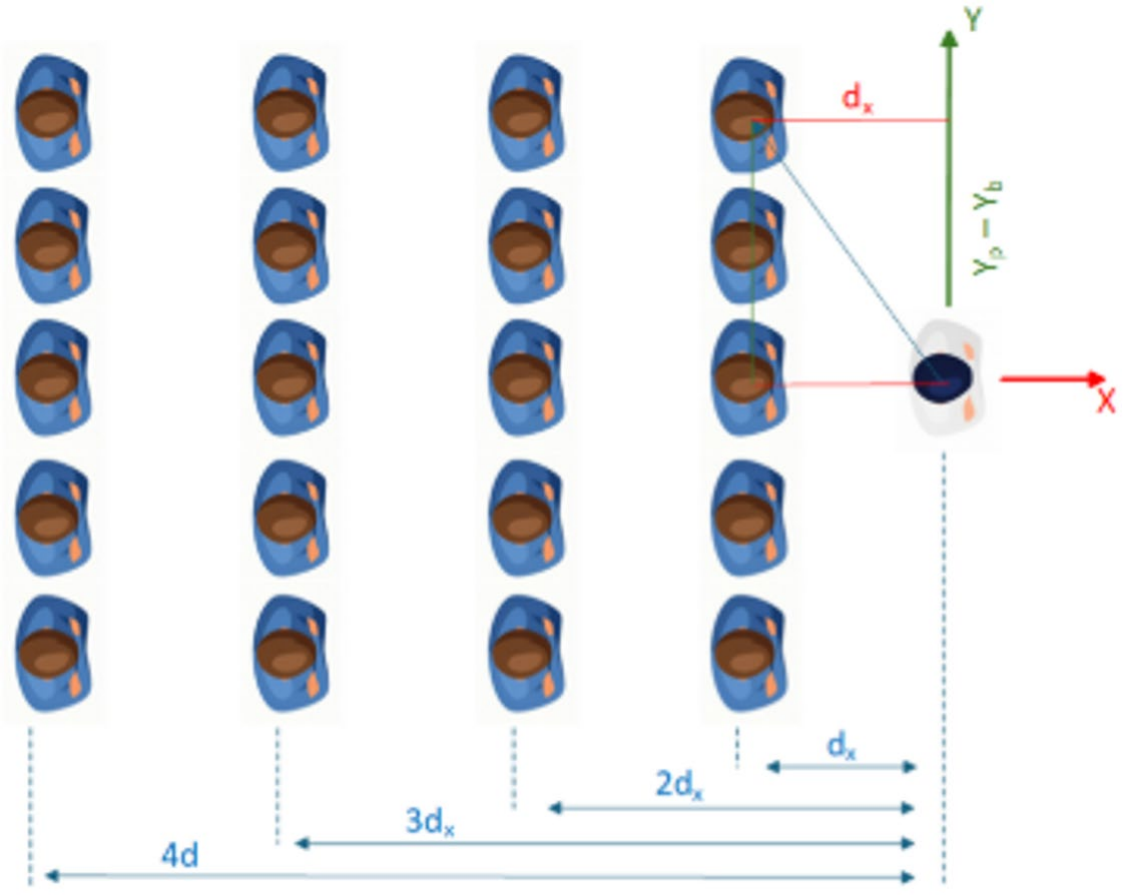
Individuals are lining up after the best solution.

In this phase individuals (prayers) are lining up in lines after Imam (best solution), the distance between each line is fixed (d) as shown in Fig. 3.

Prayers in the first lines (closest to the best solution) have higher score than who’s in the latest lines, so the individuals trying to pray at the first line.

#### Phase III: cumulative score

In Islamic pray, as individual pray more as he has higher score, and every time pray in the first line have more opportunity of having more score and being closer to the best solution, so we have to add third phase to simulate this stage of scoring behavior.

### Mathematical models

In this subsection, details the theoretical principles and mathematical models governing the proposed algorithm are explained.

#### Initialization

In general, an optimization problem contains a set of decision variables, constraints, and an objective function. It may be assumed that the number of decision variables is *\:d*, and the lower bound of the variables $\:l=[{l}_{1},\:\:{l}_{2},\:\dots\:\:{l}_{d}]$, and the upper bound is $\:u=[{u}_{1},\:\:{u}_{2},\:\dots\:\:{u}_{d}]$. Assuming that the number of prayers (population size) of POA is *\:n*. and the maximum number of iterations to *\:T*. the position *\:X* of the prayers is calculated as.

1 $$\:{X}_{i,j}={r}_{1}.\left({u}_{j}-{l}_{j}\right)+{l}_{j}\:,\:\left\{\begin{array}{c}i=1,\:2,\dots\:n\\\:j=1,\:2,\dots\:d\end{array}\right.$$

Where, $\:{r}_{1}$ is a random number uniformly distributed from 0 to 1, $\:{X}_{i,j}$ represents the *\:jth* variable of the *\:ith* individual, the detailed population information of *\:X* is

2 $$X_{{i,j}} = \left[ {\begin{array}{*{20}c} {X_{{1,1}} } & {X_{{1,2}} } & \cdots & {X_{{1,d}} } \\ {X_{{2,1}} } & {X_{{2,2}} } & \cdots & {X_{{2,d}} } \\ \vdots & \vdots & \ddots & \vdots \\ {X_{{n,1}} } & {X_{{n,2}} } & \cdots & {X_{{n,d}} } \\ \end{array} } \right],\left\{ {\begin{array}{*{20}c} {i = 1,\:2, \ldots \:n} \\ {j = 1,\:2, \ldots \:d} \\ \end{array} } \right.$$

When we assume the objective function to be $\:F\left(\:.\right)$, the fitness value for each individual is

3 $$\:{f}_{i}=F\left({X}_{i}\right)\:,i=1,\:2,\dots\:n$$

Where $\:{f}_{i}$ denotes the fitness value of bringing $\:{X}_{i}$ into the objective function $\:F\left(\:.\right)$.

#### Phase I: searching and moving to the suitable mosque

Each individual is trying to reach the most suitable mosque, according to time. When individuals have time, they will move to the farthest mosque to get higher scores, but at the last iterations each individual moves to the closest mosque. On the other hand, this phase is not just depending on the global best solution, it also balances the individual’s memory and the global best using orientation (steering) vector. It’s defined as

4 $$\:{X}_{i}^{Ref}\left(\mathrm{t}\right)=\left(1-\alpha\:\right)\:.\:\:{X}_{i}^{memory}\left(t\right)+\alpha\:\:.\:{X}^{\mathrm{*}}\left(t\right)$$

Where, $\:{X}_{i}^{Ref}$ is the guidance position vector for the *\:ith* individual, $\:{X}_{i}^{memory}$ is the historical personal best position of individual *\:i*. $\:{X}^{\mathrm{*}}\left(t\right)$ is the global best position found so far. And $\:\alpha\:$ is the interpolation factor, where

5 $$\:\alpha\:=\frac{t}{T}\:,\:i=1,\:2,\:\dots\:T$$

The number of mosques *\:m* is determined dynamically based on the problem dimension d as follow

6 $$\:m=\left\{\begin{array}{c}d\:\:\:\:\:\:\:\:\:\:\:\:\:\:\:\:\:\:\:\:\:\:\:\:\:\:\:if\:d<10\\\:round\left(\frac{d}{10}\right)\:\:\:\:\:\:if\:d\ge\:10\end{array}\right.$$

In our proposed algorithm, we identify the top *\:m* solutions as “Mosques”. These agents update their position based on their guidance vector, and depending on the distance of the mosque. The position of the mosque is defined as:

7 $$\:{X}_{m,i}\left(t\right)={X}_{m,i}\left(t\right)+{r}_{2}\:.\:D\left(t\right)\:.\:\left|{X}_{i}^{Ref}\left(t\right)-{X}_{m,i}\left(t\right)\right|$$

Where, $\:{X}_{m,i}$ the position of the *\:ith* mosque, $\:{r}_{2}$ is a random number uniformly distributed from 0 to 1, $\:D\left(t\right)$ is the mosque distance regulator (linearly decreasing from 2 to 0.2), as shown in Eq. (8).

8 $$\:D\left(t\right)=2-1.8\left(\frac{t-1}{T-1}\right),\:\:t=1,\:2,\dots\:T$$

#### Phase II: Prayer’s update

To mimic the process of lining the prayers up after Imam, the next step of our algorithm is calculating the average of the mosques positions and uses it to update positions of the rest of the population (the prayers). as:

9 $$\:{X}_{avg}\left(t\right)=\frac{1}{m}\:\sum\:_{j=1}^{m}{X}_{m,j}\left(t\right)$$

This averaged position represents the Imam-guidance point. Then, the update of prayers positions after Imam ( $\:{X}_{avg}\left(t\right)$) is calculated in Eq. (10) using the Euclidean distance as:

10 $$\:{X}_{i}^{pr}\left(t\right)={X}_{avg}\left(t\right)+{r}_{3}\:.\:\sqrt{{\left({X}_{k}^{Ref}\left(t\right)-{X}_{avg}\left(t\right)\right)}^{2}+{\delta\:}^{2}}$$

Where, $\:{X}_{k}^{Ref}\left(t\right)$ is the guidance vector of a randomly selected individual *\:k* $\:\left(k=\left[1,\:2\:,\dots\:n-m\right]\right)$, $\:{r}_{3}$ is a vector of *\:d* columns consisting of random numbers from *\:0* to *\:1*, and $\:\delta\:$ represent the spacing factor and it is calculated as:

11 $$\:\delta\:=\left(1-r\right)\:.\:{d}_{x}$$

Where, $\:{d}_{x}=1.5$, is the distance between each line of prayers.

#### Phase III: Cumulative prayer score

In Islam, the reward for prayer can not be precisely determined or calculated like any other measurable things, rather it depends on many factors, such as the degree of the worshipper’s devotion and sincerity during the pray, furthermore, these factors increase with each pray. To simplify this mathematically in our algorithm, we will apply final cumulative global update to the entire population based on a cosine oscillation, this is defined as:

12 $$\:{X}_{i}^{C}\left(t\right)={X}_{i}^{Ref}\left(t\right)+C\left(t\right)\:.\:\left(1-\frac{t}{T}\right)\:.\:\mathrm{cos}\left(\theta\:\right)\:.\:\left({X}_{i}^{Ref}\left(t\right)-{X}_{i}^{pr}\left(t\right)\right)$$

Where $\:\mathrm{cos}\left(\theta\:\right)$ introduces stochastic oscillation to help escape local optima, $\:\theta\:$ is a random number between *\:0* and $\:2\pi\:$, and $\:C\left(t\right)$ is the cumulative score regulator (linearly increasing from *\:0* to *\:2*), and it is defined as:

13 $$\:C\left(t\right)=0.2+1.8\left(\frac{t-1}{T-1}\right)\:,\:\:t=1,\:2,\dots\:T$$

#### Update the population

As a result of the search mechanism, candidate solutions converge toward promising regions of the search space. However, stochastic updates may occasionally cause decision variables to violate their prescribed limits. To ensure the feasibility of these solutions, a standard boundary-handling technique is implemented, as defined in Eq. (14).

14 $$\:{X}_{i,j}^{new}\left(t\right)=\left\{\begin{array}{c}{u}_{j}\:if\:{X}_{i,j}^{C}\left(t\right)>{u}_{j}\\\:{l}_{j}\:if\:{X}_{i,j}^{C}\left(t\right)\le\:{l}_{j}\end{array}\right.\:,\:\left\{\begin{array}{c}i=1,\:2,\dots\:n\\\:j=1,\:2,\dots\:d\end{array}\right.$$

Upon ensuring solution feasibility through boundary handling, the fitness value of each individual is computed. Subsequently, the population is updated based on the new candidate solution defined in Eq. (15).

15 $$\:{X}_{i}\left(t+1\right)=\left\{\begin{array}{c}{X}_{i}^{new}\left(t\right)\:if\:F\left({X}_{i}^{new}\left(t\right)\right)>F\left({X}_{i}\left(t\right)\right)\\\:{X}_{i}\left(t\right)\:\:\:\:if\:F\left({X}_{i}^{new}\left(t\right)\right)\le\:F\left({X}_{i}\left(t\right)\right)\end{array}\right.,\:\left\{\begin{array}{c}i=1,\:2,\dots\:n\\\:t=1,\:2,\dots\:T-1\end{array}\right.$$

The procedures outlined above constitute a single iteration of the POA algorithm. The search process terminates when the current iteration $\:t\:$reaches the maximum number of iterations *\:T*. Upon completion, the optimal individual *\:o* is identified from the final population as:

16 $$\:{X}^{\mathrm{*}}={argmin}_{1<i<n}F\left({X}_{i}\left(T\right)\right)$$

POA mimics the rituals of Islamic congregational pray. The optimization process is executed through three distinct stages: navigating to a suitable mosque, aligning in rows behind the Imam, and computing the cumulative prayer score. The detailed implementation is outlined in the pseudocode provided in Algorithm 1.

**Algorithm 1:**
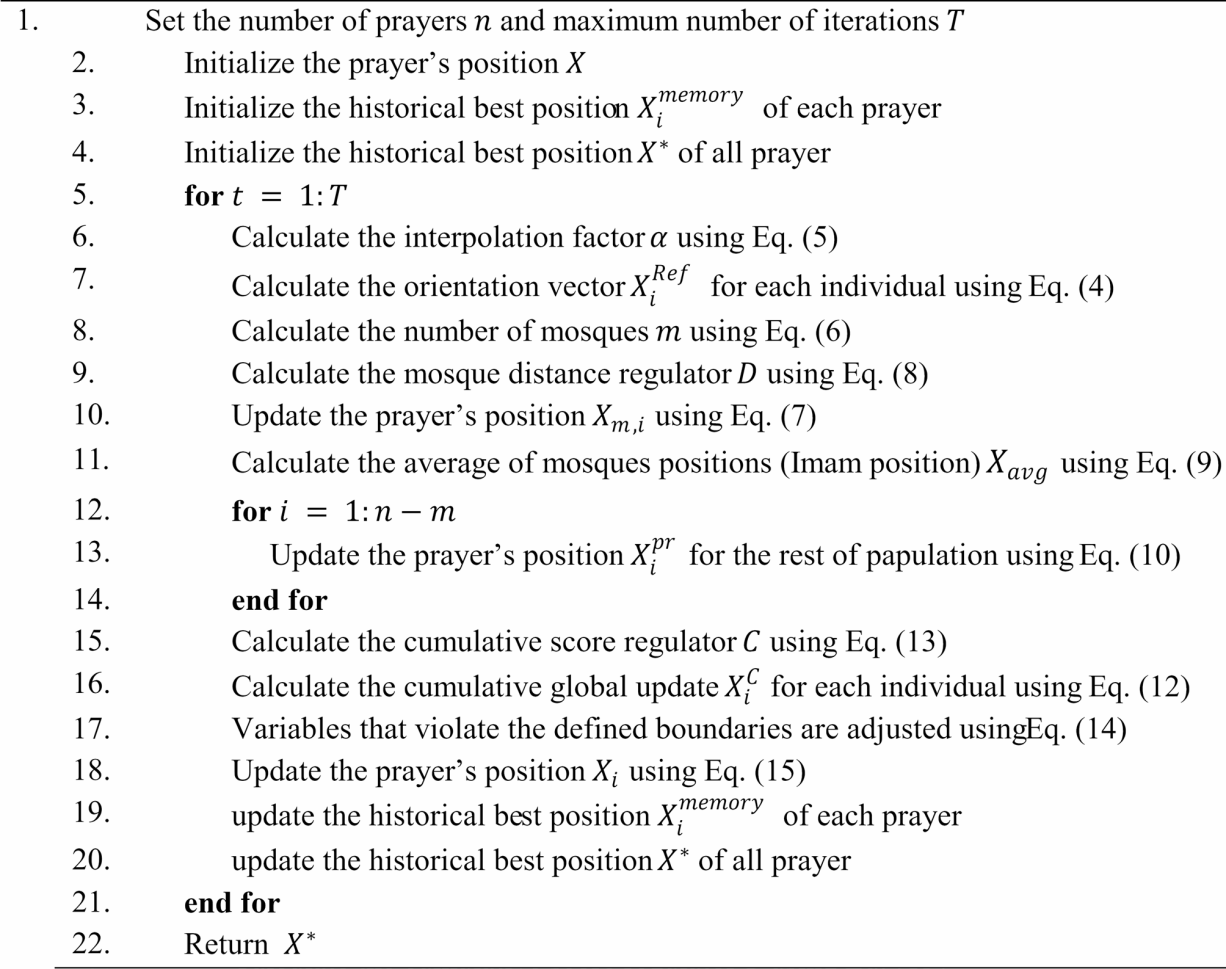
Pray Optimization Algorithm.

**Fig. 4:**
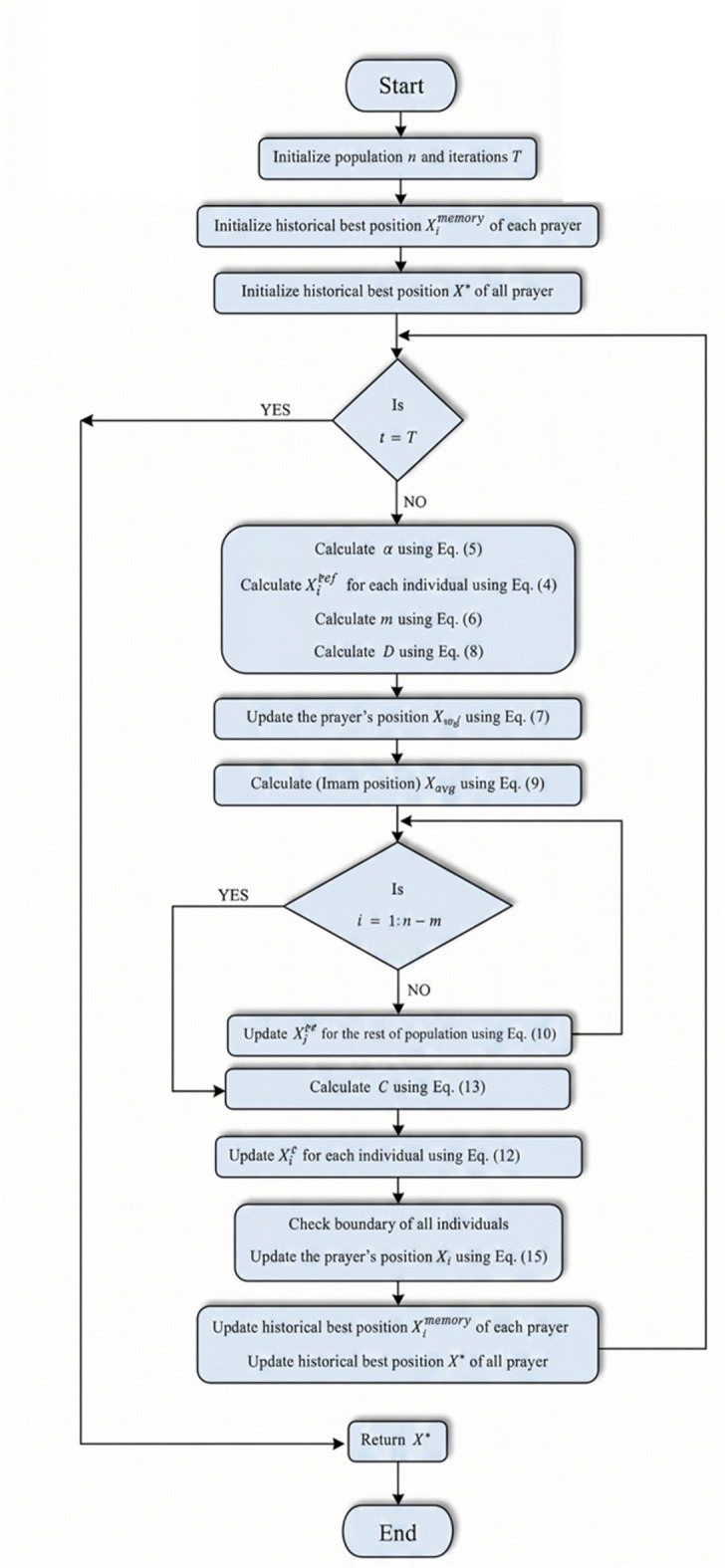
Flowchart of POA.

The Pray Optimization Algorithm (POA) begins by initializing a population of *\:n* search agents (prayers) alongside their individual and global historical best positions over a defined maximum number of iterations *\:T*, During each iteration, the algorithm progresses through a structured sequence of positional updates. First, it calculates an interpolation factor and an orientation vector, determining a dynamic subset of agents designated as “mosques” *\:m* and updating their coordinates using a specific distance regulator. Following this, the algorithm computes the average position of these mosques to establish a central “Imam position” $\:{X}_{avg}$, which serves as a spatial reference to update the remaining *\:n-m* agents in the population. Finally, a cumulative score regulator *\:C* is calculated to execute a global positional update for every individual. At the end of each iteration, any variables that violate the search space boundaries are corrected, and both the individual memories $\:{X}_{i}^{memory}$ and the global best solution $\:{X}^{\mathrm{*}}$ are updated before the cycle repeats, and this is illustrated visually in the flowchart shown at Fig. 4.

Parameters of the proposed algorithm are shown in Table 1.

**Table 1:** Parameters of Pray Optimization Algorithm.

Parameter	Symbol	Rang	description
Interpolation factor	$\:\alpha\:$	$\:0\:to\:1$	Balancing agent memory Vs. global leader.
Mosque distance	$\:D\left(t\right)$	$\:2\:to\:0.2$	Controls the step size of mosques (Exploration).
Cumulative score	$\:C\left(t\right)$	$\:0.2\:to\:2$	Controls magnitude of final perturbation (Exploitation).
Line spacing	$\:{d}_{x}$	1.5	Constant determining spacing between prayers lines.

### Computational complexity

Computational complexity serves as a fundamental metric for assessing algorithmic efficiency. Consequently, this subsection provides a detailed analysis of both the time and space complexity of the proposed POA.

#### Time complexity

The time complexity of the POA is determined by the computational cost of the initialization phase and the cumulative cost of the iterative search mechanism. Let *\:n* denote the population size, *\:T* the maximum number of iterations, and *\:d* the dimensionality of the problem. During initialization, generating the initial population and evaluating the fitness of *\:n* individuals require $\:O\left(n\times\:d\right)$ time. Subsequently, the algorithm executes a loop *\:T* times, where each iteration comprises three phases: updating mosque positions (Phase I), aligning prayers (Phase II), and calculating the cumulative score (Phase III). Additionally, the calculation of the guidance vector and boundary handling are performed within each loop. Since these steps involve vector arithmetic operations applied to the entire population across *\:d* dimensions, the computational cost per iteration is $\:O\left(n\times\:d\right)$. Therefore, the total time complexity over *\:T* iterations is $\:O\left(T\times\:n\times\:d\right)$ resulting in a final complexity of $\:O\left(Tnd\right)$.

#### Space complexity

In terms of space complexity, the memory requirements of the POA are dictated by the data structures necessary to maintain solution information throughout the optimization process. The algorithm requires the allocation of memory for the current population matrix, the historical personal best (memory) matrix, and temporary variables such as the guidance vectors. Each of these structures stores position data for *\:n* individuals across *\:d* dimensions, resulting in a size of $\:n\times\:d$. Auxiliary variables and scalar parameters occupy negligible space relative to the population matrices. Consequently, the overall space complexity of the proposed algorithm is $\:O\left(nd\right)$.

### Operator-level comparison with existing metaheuristics

To further clarify the novelty of the proposed POA, this subsection provides an operator-level comparison between POA and representative swarm and evolutionary algorithms, including PSO, BBBC, SCA, GWO, WOA, and HHO. Most conventional leader-based algorithms, such as PSO, GWO, WOA, and HHO, rely on direct position updates driven by one or more reference solutions (e.g., global best, alpha agents, or prey positions) through vector addition or weighted attraction mechanisms. Similarly, BBBC-based methods use a centroid operator to collapse the population toward a single representative point, while sine-cosine-based methods introduce oscillatory motion directly on individual positions without altering the underlying population structure.

In contrast, POA employs a structured multi-stage operator design. First, a collective guidance point is constructed using the centroid of the top-performing agents. Second, unlike conventional direct displacement updates, POA introduces a geometric positioning mechanism that assigns individuals into structured formations relative to this reference point. Finally, a bounded stochastic modulation is applied to these structured positions to refine exploration and exploitation behavior. Therefore, the key distinction of POA lies not in the presence of individual operators such as leader guidance, centroid computation, or stochastic perturbation, but in the sequential integration of these components within a structured population formation framework that defines how information is propagated through the search process. Table 2 presents comparison of Search Operators in POA and Existing Metaheuristic Algorithms.

**Table 2:** Operator level comparison of POA and representative metaheuristic algorithms.

Algorithm	Guidance source	Update type	Population structure
PSO	pbest + gbest	Velocity update	Unstructured
GWO	alpha/beta/delta	Weighted position	Unstructured
WOA	prey	Encircling spiral	Unstructured
BBBC	centroid	Collapse to mean	Fully collapsed
SCA	random + sine/cosine	Oscillation	Unstructured
POA	top-m centroid	Structured spatial assignment + stochastic refinement	Structured formation

## Experimental results and discussion

This section presents the experimental results of the POA on 27 benchmark functions, analyzing its optimization capabilities, convergence speed, scalability, and statistical tests of POA.

### Benchmark functions and comparison algorithms

To rigorously evaluate the global exploration, local exploitation, and local optima avoidance capabilities of the POA, the CEC2017^21^ benchmark test suite was employed.

**Table 3:** Summary of the CEC2017 benchmark functions^27^.

	No	Function	Range	$\:{F}_{best}$
Unimodal functions	$\:{F}_{1}$	Shifted and Rotated Bent Cigar Function	[-100,100]	100
	$\:{F}_{3}$	Shifted and Rotated Bent Zakharov Function	[-100,100]	300
Multimodal functions	$\:{F}_{6}$	Shifted and Rotated Expanded Scaffer’s F6 Function	[-100,100]	600
	$\:{F}_{7}$	Shifted and Rotated Lunacek Bi_Rastrigin Function	[-100,100]	700
	$\:{F}_{8}$	Shifted and Rotated Non-Continuous Rastrigin’s Function	[-100,100]	800
	$\:{F}_{9}$	Shifted and Rotated Levy Function	[-100,100]	900
	$\:{F}_{10}$	Shifted and Rotated Schwefel’s Function	[-100,100]	1000
Hybrid functions	$\:{F}_{11}$	Hybrid Function 1 $\:(N\:=\:3)$	[-100,100]	1100
	$\:{F}_{12}$	Hybrid Function $\:2\:(N\:=\:3)$	[-100,100]	1200
	$\:{F}_{13}$	Hybrid Function $\:3\:(N\:=\:3)$	[-100,100]	1300
	$\:{F}_{14}$	Hybrid Function $\:4\:(N\:=\:4)$	[-100,100]	1400
	$\:{F}_{15}$	Hybrid Function $\:5\:(N\:=\:4)$	[-100,100]	1500
	$\:{F}_{16}$	Hybrid Function $\:6\:(N\:=\:4)$	[-100,100]	1600
	$\:{F}_{17}$	Hybrid Function $\:6\:(N\:=\:5)$	[-100,100]	1700
	$\:{F}_{18}$	Hybrid Function $\:6\:(N\:=\:5)$	[-100,100]	1800
	$\:{F}_{19}$	Hybrid Function $\:6\:(N\:=\:5)$	[-100,100]	1900
	$\:{F}_{20}$	Hybrid Function $\:6\:(N\:=\:6)$	[-100,100]	2000
Composition functions	$\:{F}_{21}$	Composition Function $\:1\:(N\:=\:3)$	[-100,100]	2100
	$\:{F}_{22}$	Composition Function $\:2\:(N\:=\:3)$	[-100,100]	2200
	$\:{F}_{23}$	Composition Function $\:3\:(N\:=\:4)$	[-100,100]	2300
	$\:{F}_{24}$	Composition Function $\:4\:(N\:=\:4)$	[-100,100]	2400
	$\:{F}_{25}$	Composition Function $\:5\:(N\:=\:5)$	[-100,100]	2500
	$\:{F}_{26}$	Composition Function $\:6\:(N\:=\:5)$	[-100,100]	2600
	$\:{F}_{27}$	Composition Function $\:7\:(N\:=\:6)$	[-100,100]	2700
	$\:{F}_{28}$	Composition Function $\:8\:(N\:=\:6)$	[-100,100]	2800
	$\:{F}_{29}$	Composition Function 9$\:\:(N\:=\:3)$	[-100,100]	2900
	$\:{F}_{30}$	Composition Function 10$\:\:(N\:=\:3)$	[-100,100]	3000

#### Description of the benchmark functions

The CEC2017 benchmark suite effectively simulates the complex fitness landscapes characteristic of real-world optimization problems, providing a highly challenging testing environment. To comprehensively evaluate the performance and versatility of the Prayer Optimization Algorithm (POA) across diverse problem topologies, our experimental study incorporates 29 functions from this suite. Table 3 shows the specific information of the CEC2017 suite.

- Unimodal Functions $\:({F}_{1},\:{F}_{3})$ Characterized by a single global optimum and the absence of local minima, these functions are primarily utilized to evaluate an algorithm’s exploitation capability. Their evaluative focus centers on convergence speed and the precision of the final solution.
- Multimodal Functions $\:({F}_{4}-\:{F}_{10})$ Featuring a multitude of local optima, these functions assess an algorithm’s exploration capability. To succeed, an algorithm must demonstrate not only strong convergence properties but also the ability to effectively escape local traps to locate the global optimum.
- Hybrid Functions $\:({F}_{11}-\:{F}_{20})$ Constructed by aggregating multiple basic functions and applying transformations such as rotation and translation, hybrid functions are designed to simulate real-world optimization problems that possess diverse substructures and complex fitness landscapes.
- Composition Functions $\:({F}_{21}-\:{F}_{30})$ By embedding various basic functions into a unified search space via shifting, scaling, and rotation, composition functions simulate highly deceptive environments. They are specifically formulated to test an algorithm’s performance across landscapes characterized by regional heterogeneity and discontinuous properties.

#### Comparison algorithms

To comprehensively evaluate the performance of the proposed POA, six prominent metaheuristic algorithms were selected for comparison. Particle Swarm Optimization (PSO)^9^ was chosen as a classic, foundational benchmark. The Whale Optimization Algorithm (WOA)^14^, Harris Hawks Optimization (HHO)^13^, and Crayfish Optimization Algorithm (COA)^28^ were included as highly regarded representatives of swarm intelligence. Additionally, Rüppell’s Fox Optimizer (RFO)^29^ was selected to benchmark against a recently proposed method, while the Arithmetic Optimization Algorithm (AOA)^30^ was included to represent mathematics-based approaches.

Table 4 details the parameter configurations for all evaluated algorithms. To ensure a fair comparison, the control parameters for the competing algorithms were set strictly according to their original publications. Furthermore, to mitigate the influence of stochasticity, each algorithm was independently executed 51 times on every benchmark function. The mean fitness value (Mean) and standard deviation (Std) across these 51 independent runs were recorded as the primary performance metrics.

**Table 4:** Parameter settings of the proposed POA algorithm and other competing algorithms.

Algorithm	Parameter	Value
POA	*\:D*	[2 to 0.2]
	*\:C*	[0.2 to 2]
PSO^9^	$\:{C}_{1}$	2.0
	$\:{C}_{2}$	2.0
	$\:{\omega\:}_{min}$	0.4
	$\:{\omega\:}_{max}$	0.9
WOA^14^	$\:\overrightarrow{a}$	[2 to 0.2]
	$$\:\overrightarrow{A}$$	[-2, 2]
	$$\:\overrightarrow{C}$$	[0, 2]
HHO^13^	$\:{E}_{0}$	[-1 to 1]
RFO^29^	$\:\beta\:$	0.000001
	$\:{e}_{0},\:{e}_{1}$	1, 3
	$\:{C}_{0},\:{C}_{1}$	2, 2
	$\:{a}_{0},\:{a}_{1}$	2, 3
COA^28^	$\:{C}_{0}$	0.2
	$\:{C}_{1}$	3
	$\:\mu\:$	25
	$\:\sigma\:$	3
AOA^30^	$\:\alpha\:$	5
	$\:\mu\:$	0.5
	Mop-max	1
	Mop_min	0.2

#### Experimental setup

To evaluate the optimization performance and verify the scalability of the proposed POA, experiments were conducted across multiple dimensions: $\:D\:=\:10,\:30,\:50,\:and\:100$. A moderate dimensionality of $\:D\:=\:50$ was established as the primary baseline for the benchmark testing. For all experiments, the population size was set to 50, and the termination criterion was defined by a maximum number of function evaluations $\:MaxFEs\:equal\:to\:\mathrm{10,000}\:\times\:\:D\:\:$(e.g., $\:MaxFEs=5\:\times\:\:{10}^{5}$ evaluations for $\:D\:=\:50$). All simulations for the POA and the comparative algorithms were executed on a 64-bit Ubuntu 22.04 LTS operating system equipped with an Intel Core i5-5200U CPU and 8 GB of RAM, utilizing MATLAB R2023b.

### Qualitative analysis

A qualitative analysis is conducted in this subsection to visually investigate the convergence behavior and spatial distribution of the POA over successive iterations. The parameter settings for this demonstration are fixed at a dimensionality of $\:D\:=\:2$, a population size of *\:50*, and a maximum iteration count of $\:T\:=\:500$.

**Fig. 5:**
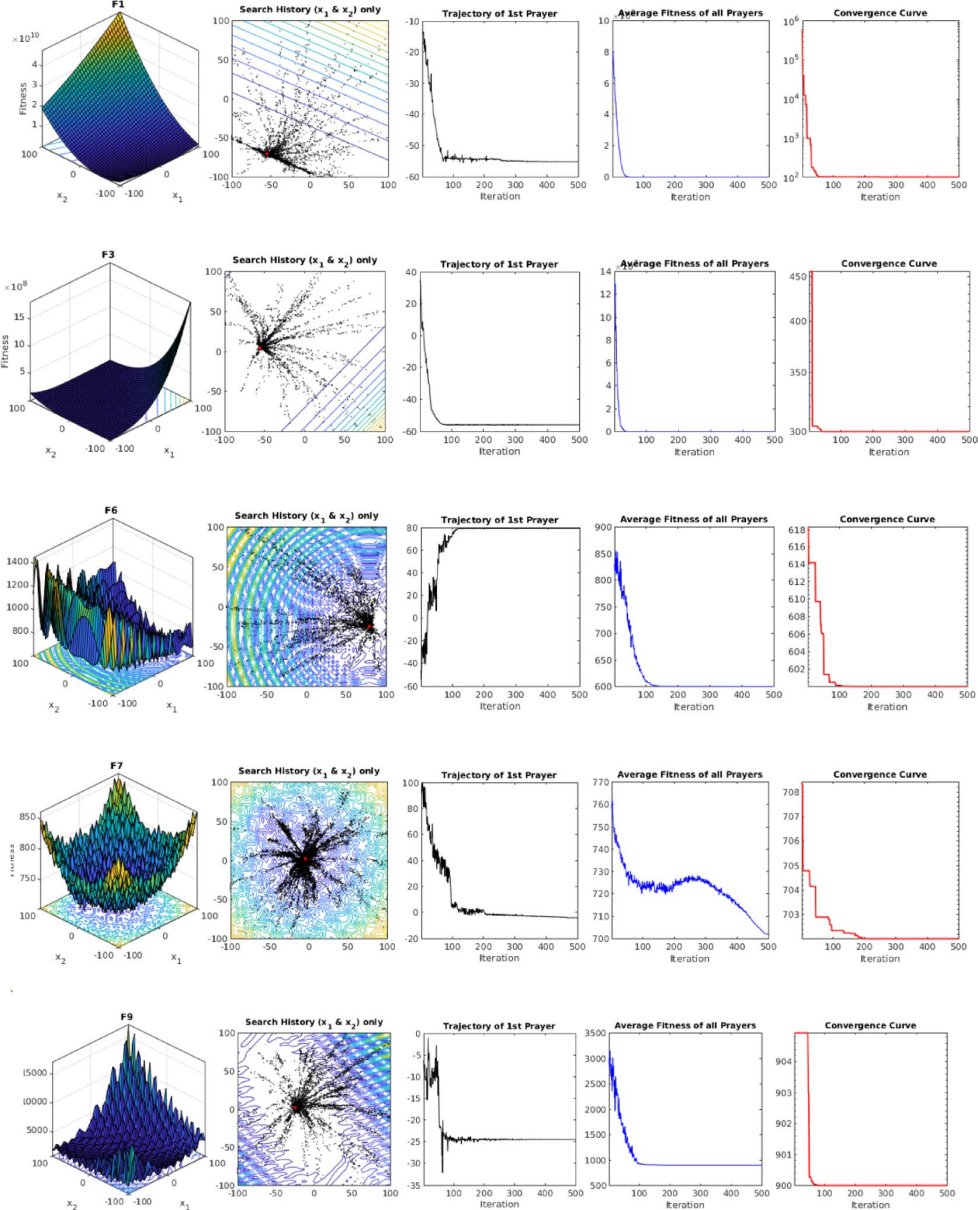

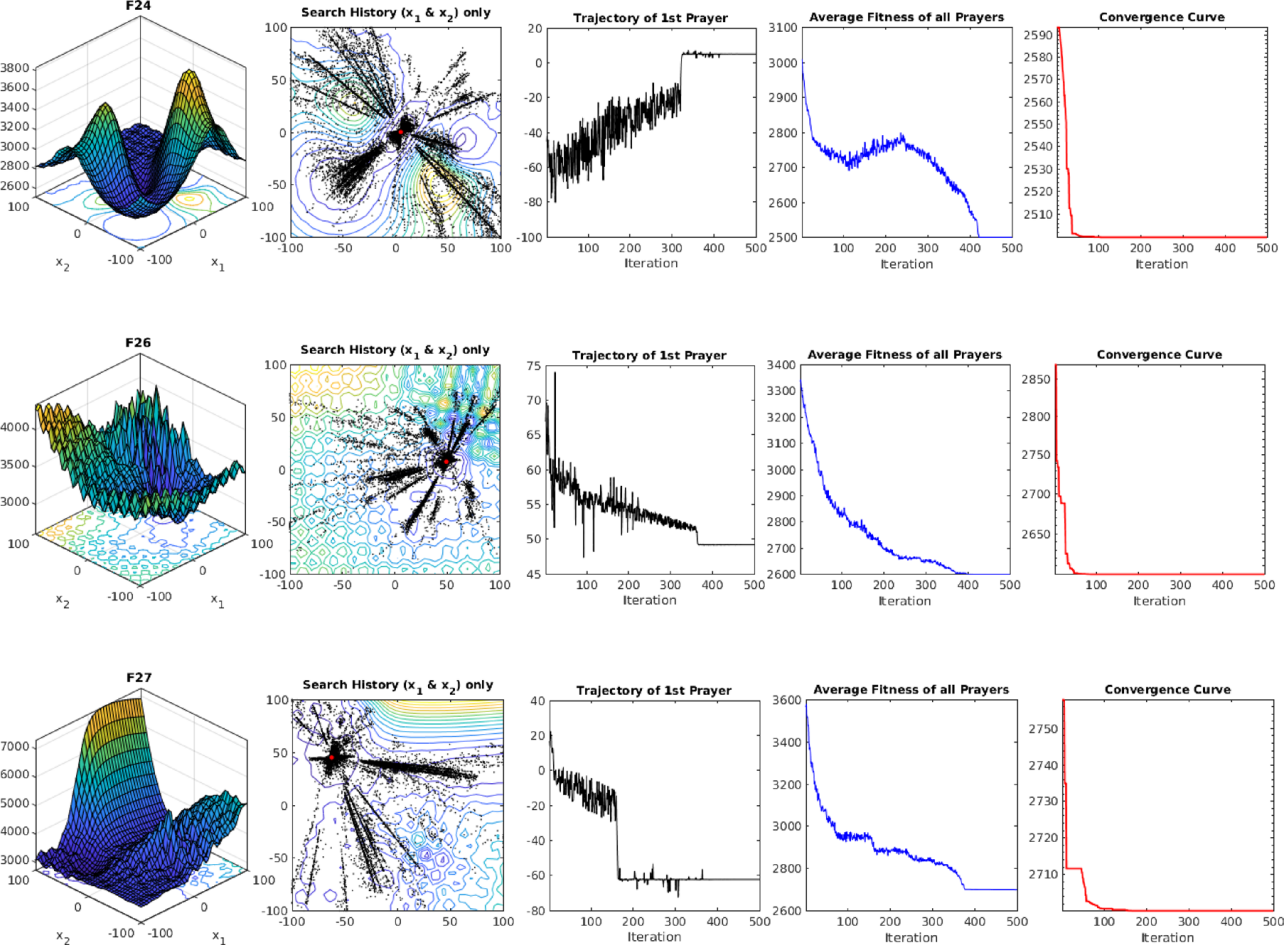
The 2D landscape, search history, trajectories, average fitness, and convergence curves of some functions.

Figure 5 presents a qualitative analysis of the POA across a representative subset of the CEC2017 benchmark suite, encompassing two unimodal, three multimodal, and three composition functions. The figure is organized into five analytical columns: the first column illustrates the 2D topological landscape of the objective functions. The subsequent four columns respectively depict the spatial search history of the agents, the one-dimensional search trajectory of the first agent, the evolution of the population’s average fitness, and the global convergence curve.

The qualitative metrics presented in Fig. 5 validate the dynamic balance between exploration and exploitation within the POA. By plotting the historical positions of the search agents, the second column confirms the POA’s global search efficacy. The algorithm’s robust exploitation capability is evidenced by the dense clustering of agents around the final global optimum (indicated by the red marker). Crucially, the agents exhibit a broader scattering pattern on complex multimodal and composition functions compared to unimodal ones, highlighting the algorithm’s adaptive capacity to balance localized exploitation with the global exploration required to evade local optima.

The ‘Trajectory of 1st Prayer’ exhibits high-frequency, large-amplitude oscillations during the early iterations. This confirms that the algorithm aggressively searches diverse regions before smoothly dampening into a flat line, which signifies a successful transition from exploration to localized exploitation. The ‘Average Fitness of all Prayers’ curves display a rapid, continuous decline, proving that the entire population collectively improves and converges toward promising regions without diverging. Finally, the ‘Convergence Curve’ for each function exhibits a steep initial drop followed by a stable plateau, validating the POA’s fast convergence speed and its ability to efficiently locate optimal or near-optimal solutions.

### Statistical analysis

To evaluate the general optimization performance of POA, this section tests the proposed POA algorithm and six comparison algorithms on 50-dimensional CEC2017 benchmark functions. The statistical results are shown in Table 5. Considering the overall results across the evaluated functions, POA achieved the 2st rank same as PSO with a Friedman Mean Rank (FMR) of 2.52. For the unimodal function $\:{F}_{1}$, POA shows superior optimization accuracy and stability compared to the other algorithms, obtaining the best mean fitness value, standard deviation, and the theorical optimal fitness $\:\left(100\right)$ as the best minimum. Among the simple multimodal functions $\:({F}_{4}\:\sim\:{F}_{10}),$ POA exhibits highly competitive performance. It secures the second optimal mean results on functions $\:{F}_{4},\:{F}_{5},\:{F}_{7},\:{F}_{8},\:{F}_{9},\:and\:{F}_{10}$. On functions $\:{F}_{4}\:and\:{F}_{9}$ specifically, POA obtains the best std indicator outperforming the other algorithms. For the hybrid functions, POA demonstrates excellent exploitation capabilities, on the $\:{F}_{11},\:{F}_{12},\:{F}_{13},\:and\:{F}_{14}\:$functions, POA achieves better results than the other algorithms in almost all statistical metrics. On the $\:{F}_{15},\:{F}_{18},\:{F}_{19},\:and\:{F}_{20}\:$function, POA scores the second-best mean fitness value. Furthermore, POA has better std indexes on most of these complex hybrid functions, ensuring stable convergence. Among the composition functions, POA outperforms the other algorithms on $\:{F}_{28}$, securing the **best** mean and second-best std values. In addition to this, POA acquires suboptimal mean results on the$\:\:{F}_{22}\:and\:{F}_{25}\:\:$composition functions.

**Table 5:** Optimization results for POA and its comparison algorithms on CEC2017 benchmark functions run independently 51, and the number of variables is 50. (The best result is marked in bold, and italics indicate that the algorithm achieves suboptimal results.)

NO	Metrics	POA	PSO	WOA	HHO	RFO	COA	AOA
F1	Mean	**1.57e + 03**	7.73e + 09	*8.87e + 06*	3.40e + 07	8.69e + 10	1.59e + 07	1.02e + 11
	Std	**1.88e + 03**	7.08e + 09	7.11e + 06	*6.19e + 06*	1.35e + 10	1.00e + 08	9.47e + 09
	Best	**1.00e + 02**	8.74e + 08	1.09e + 06	1.95e + 07	6.08e + 10	*1.26e + 04*	7.02e + 10
	Worst	**1.02e + 04**	3.48e + 10	*3.40e + 07*	6.39e + 07	1.26e + 11	7.16e + 08	1.18e + 11
	Time(S)	*4.5704*	22.7195	**4.4996**	11.5431	8.7129	6.4256	8.3161
	Rank	1	5	2	4	6	3	7
F3	Mean	4.03e + 04	*1.27e + 04*	8.21e + 04	**2.71e + 03**	1.35e + 05	6.25e + 04	2.14e + 05
	Std	*8.58e + 03*	1.05e + 04	3.49e + 04	**7.71e + 02**	2.18e + 04	2.25e + 04	3.64e + 04
	Best	2.19e + 04	*2.92e + 03*	3.82e + 04	**1.45e + 03**	6.79e + 04	2.81e + 04	1.45e + 05
	Worst	5.85e + 04	*4.52e + 04*	1.92e + 05	**4.68e + 03**	1.71e + 05	1.23e + 05	3.00e + 05
	Time(S)	**5.0132**	23.3297	*5.1194*	12.4763	9.2210	7.0516	9.4554
	Rank	3	2	5	1	6	4	7
F4	Mean	*5.75e + 02*	**1.22e + 03**	6.80e + 02	6.19e + 02	1.71e + 04	5.36e + 02	3.81e + 04
	Std	**3.82e + 01**	8.17e + 02	5.36e + 01	6.03e + 01	5.49e + 03	*5.09e + 01*	7.43e + 03
	Best	5.29e + 02	*5.56e + 02*	5.09e + 02	**4.34e + 02**	5.48e + 03	**4.34e + 02**	1.99e + 04
	Worst	*6.77e + 02*	**4.72e + 03**	8.06e + 02	7.48e + 02	3.22e + 04	6.39e + 02	5.46e + 04
	Time(S)	*4.6688*	21.7255	**4.5499**	11.5136	8.6937	6.5553	8.8439
	Rank	2	5	4	3	6	1	7
F5	Mean	*8.35e + 02*	**6.61e + 02**	9.19e + 02	8.72e + 02	1.00e + 03	8.71e + 02	1.27e + 03
	Std	3.87e + 01	*2.75e + 01*	7.31e + 01	3.25e + 01	5.22e + 01	**2.29e + 01**	5.81e + 01
	Best	*7.48e + 02*	**6.12e + 02**	8.06e + 02	8.11e + 02	8.53e + 02	8.13e + 02	1.10e + 03
	Worst	*9.19e + 02*	**7.11e + 02**	1.12e + 03	9.61e + 02	1.12e + 03	9.22e + 02	1.36e + 03
	Time(S)	**5.5363**	25.0064	*5.7789*	14.6248	10.1403	8.2215	10.0501
	Rank	2	1	5	4	6	3	7
F6	Mean	6.57e + 02	**6.06e + 02**	6.77e + 02	6.68e + 02	6.65e + 02	*6.54e + 02*	7.18e + 02
	Std	*3.98e + 00*	**2.75e + 00**	8.55e + 00	4.39e + 00	8.40e + 00	1.47e + 01	9.48e + 00
	Best	6.50e + 02	**6.01e + 02**	6.58e + 02	6.57e + 02	6.47e + 02	6.15e + 02	6.95e + 02
	Worst	*6.68e + 02*	**6.13e + 02**	7.02e + 02	6.79e + 02	6.83e + 02	6.72e + 02	7.40e + 02
	Time(S)	**8.5569**	25.8955	*8.6398*	21.6634	12.5491	14.0007	12.7481
	Rank	3	1	6	5	4	2	7
F7	Mean	*1.58e + 03*	**9.46e + 02**	1.67e + 03	1.74e + 03	1.85e + 03	1.65e + 03	2.17e + 03
	Std	1.27e + 02	*6.51e + 01*	1.23e + 02	9.31e + 01	1.79e + 02	1.43e + 02	**5.39e + 01**
	Best	*1.28e + 03*	**8.52e + 02**	1.39e + 03	1.51e + 03	1.53e + 03	1.29e + 03	2.04e + 03
	Worst	1.95e + 03	**1.21e + 03**	*1.89e + 03*	*1.89e + 03*	2.31e + 03	1.81e + 03	2.22e + 03
	Time(S)	**5.3309**	22.0557	*5.3734*	13.6676	9.4569	8.2014	9.3873
	Rank	2	1	4	5	6	3	7
F8	Mean	*1.14e + 03*	**9.62e + 02**	1.21e + 03	1.15e + 03	1.31e + 03	1.19e + 03	1.60e + 03
	Std	4.70e + 01	3.65e + 01	6.82e + 01	**3.04e + 01**	5.11e + 01	*3.63e + 01*	5.53e + 01
	Best	*1.02e + 03*	**8.88e + 02**	1.09e + 03	1.09e + 03	1.22e + 03	1.05e + 03	1.44e + 03
	Worst	*1.24e + 03*	**1.06e + 03**	1.37e + 03	1.24e + 03	1.46e + 03	1.23e + 03	1.71e + 03
	Time(S)	**5.1747**	22.4656	*5.1750*	13.1045	9.1965	7.8126	9.3487
	Rank	2	1	5	3	6	4	7
F9	Mean	*1.09e + 04*	**3.10e + 03**	2.16e + 04	1.65e + 04	2.14e + 04	1.53e + 04	5.18e + 04
	Std	**1.03e + 03**	*1.58e + 03*	7.21e + 03	1.84e + 03	3.80e + 03	2.82e + 03	8.46e + 03
	Best	8.21e + 03	**1.47e + 03**	1.23e + 04	1.22e + 04	1.38e + 04	*7.06e + 03*	3.36e + 04
	Worst	*1.29e + 04*	**8.35e + 03**	4.13e + 04	2.02e + 04	3.08e + 04	2.35e + 04	7.71e + 04
	Time(S)	**5.1762**	22.4467	*5.2735*	13.4917	8.8754	7.5449	9.4218
	Rank	2	1	6	4	5	3	7
F10	Mean	*8.26e + 03*	**6.84e + 03**	1.00e + 04	8.34e + 03	1.08e + 04	9.93e + 03	1.53e + 04
	Std	1.15e + 03	*9.25e + 02*	1.34e + 03	8.04e + 02	7.49e + 02	**7.25e + 02**	9.58e + 02
	Best	*5.69e + 03*	**4.98e + 03**	7.15e + 03	6.56e + 03	9.52e + 03	8.16e + 03	1.33e + 04
	Worst	1.08e + 04	**8.97e + 03**	1.36e + 04	*1.00e + 04*	1.28e + 04	1.11e + 04	1.78e + 04
	Time(S)	**6.8166**	25.2980	*7.0173*	17.3971	10.4630	10.1561	10.8095
	Rank	2	1	5	3	6	4	7
F11	Mean	**1.29e + 03**	1.51e + 03	1.59e + 03	*1.41e + 03*	1.10e + 04	1.43e + 03	3.76e + 04
	Std	**4.02e + 01**	1.29e + 02	9.36e + 01	*6.66e + 01*	4.18e + 03	7.18e + 01	1.38e + 04
	Best	**1.22e + 03**	1.25e + 03	1.42e + 03	*1.28e + 03*	4.83e + 03	1.30e + 03	1.91e + 04
	Worst	**1.41e + 03**	1.92e + 03	1.83e + 03	*1.54e + 03*	2.10e + 04	1.63e + 03	8.48e + 04
	Time(S)	**4.9658**	22.0251	*5.0257*	13.1897	9.0397	7.1848	9.2287
	Rank	1	4	5	2	6	3	7
F12	Mean	**7.86e + 06**	1.93e + 09	2.16e + 08	5.33e + 07	1.66e + 10	*8.80e + 06*	8.42e + 10
	Std	**3.73e + 06**	2.10e + 09	1.14e + 08	2.08e + 07	8.14e + 09	*5.06e + 06*	1.76e + 10
	Best	2.44e + 06	*1.68e + 06*	4.39e + 07	1.83e + 07	3.34e + 09	**1.26e + 06**	2.74e + 10
	Worst	**1.83e + 07**	1.03e + 10	5.85e + 08	1.13e + 08	4.29e + 10	*2.23e + 07*	1.21e + 11
	Time(S)	**5.4953**	22.5244	*5.5669*	14.2278	9.3241	8.1338	9.0203
	Rank	1	5	4	3	6	2	7
F13	Mean	**4.66e + 04**	4.81e + 08	1.76e + 05	1.40e + 06	2.66e + 09	*5.72e + 04*	4.87e + 10
	Std	**2.04e + 04**	1.03e + 09	1.12e + 05	7.68e + 05	3.28e + 09	*2.74e + 04*	1.23e + 10
	Best	*1.50e + 04*	1.61e + 04	5.18e + 04	5.47e + 05	2.52e + 07	**1.48e + 04**	2.64e + 10
	Worst	**1.05e + 05**	3.53e + 09	7.36e + 05	5.18e + 06	2.03e + 10	*1.58e + 05*	8.27e + 10
	Time(S)	**4.9883**	22.3870	*5.1366*	12.8136	8.8578	7.2898	9.2204
	Rank	1	5	3	4	6	2	7
F14	Mean	**2.64e + 04**	5.05e + 05	7.46e + 05	2.57e + 05	9.32e + 04	*8.24e + 04*	1.74e + 08
	Std	**1.60e + 04**	1.16e + 06	5.28e + 05	2.06e + 05	3.09e + 05	*6.84e + 04*	1.03e + 08
	Best	*4.74e + 03*	6.36e + 03	5.54e + 04	2.92e + 04	**1.92e + 03**	1.15e + 04	2.35e + 07
	Worst	**8.40e + 04**	7.85e + 06	2.06e + 06	9.35e + 05	2.21e + 06	*2.89e + 05*	5.01e + 08
	Time(S)	**5.9939**	24.4657	*6.2636*	15.7969	9.4341	8.8657	10.2230
	Rank	1	5	6	4	3	2	7
F15	Mean	*1.97e + 04*	2.84e + 06	8.32e + 04	1.98e + 05	3.92e + 06	**1.84e + 04**	1.32e + 10
	Std	*7.68e + 03*	1.40e + 07	6.02e + 04	9.94e + 04	1.47e + 07	**6.49e + 03**	5.22e + 09
	Best	8.55e + 03	*4.28e + 03*	1.06e + 04	4.41e + 04	6.20e + 03	**3.56e + 03**	2.14e + 09
	Worst	5.16e + 04	*7.13e + 07*	2.63e + 05	6.30e + 05	8.74e + 07	**2.59e + 04**	2.32e + 10
	Time(S)	**4.7678**	22.4648	*4.8456*	12.3911	9.1974	6.8812	8.9365
	Rank	2	5	3	4	6	1	7
F16	Mean	3.79e + 03	**3.02e + 03**	4.90e + 03	4.11e + 03	4.88e + 03	*3.10e + 03*	9.52e + 03
	Std	5.24e + 02	**3.49e + 02**	6.85e + 02	4.59e + 02	6.55e + 02	*4.00e + 02*	1.50e + 03
	Best	2.83e + 03	**2.19e + 03**	3.89e + 03	3.13e + 03	3.53e + 03	*2.22e + 03*	6.22e + 03
	Worst	5.78e + 03	**3.71e + 03**	6.77e + 03	5.39e + 03	6.08e + 03	*3.93e + 03*	1.28e + 04
	Time(S)	**5.4042**	23.4060	*5.5217*	14.2282	9.5762	8.0131	9.6536
	Rank	3	1	6	4	5	2	7
F17	Mean	4.00e + 03	**2.88e + 03**	3.99e + 03	3.57e + 03	3.37e + 03	*3.09e + 03*	6.73e + 04
	Std	*4.79e + 02*	3.14e + 02	4.66e + 02	3.79e + 02	3.15e + 02	**3.13e + 02**	4.99e + 04
	Best	3.02e + 03	**2.21e + 03**	2.88e + 03	2.66e + 03	2.68e + 03	*2.45e + 03*	1.14e + 04
	Worst	5.36e + 03	**3.64e + 03**	4.81e + 03	4.23e + 03	3.96e + 03	*3.86e + 03*	1.78e + 05
	Time(S)	**8.2825**	26.1610	*8.6415*	21.3128	12.4137	12.9609	12.4524
	Rank	6	1	5	4	3	2	7
F18	Mean	*8.09e + 05*	1.04e + 06	4.67e + 06	1.88e + 06	**4.38e + 05**	1.28e + 06	3.27e + 08
	Std	**4.74e + 05**	1.24e + 06	2.65e + 06	1.18e + 06	*5.50e + 05*	1.36e + 06	2.24e + 08
	Best	1.89e + 05	1.54e + 05	7.75e + 05	2.92e + 05	**2.82e + 04**	*7.89e + 04*	4.41e + 07
	Worst	**2.09e + 06**	8.13e + 06	1.63e + 07	5.35e + 06	*2.30e + 06*	7.52e + 06	1.01e + 09
	Time(S)	**5.4331**	25.1553	*5.6835*	14.2544	9.3947	7.9773	9.7882
	Rank	2	3	6	5	1	4	7
F19	Mean	*1.05e + 05*	1.05e + 06	2.08e + 06	5.19e + 05	7.09e + 06	**2.16e + 04**	7.12e + 09
	Std	*9.97e + 04*	2.64e + 06	1.69e + 06	3.33e + 05	1.07e + 07	**1.46e + 04**	2.43e + 09
	Best	2.02e + 04	*2.83e + 03*	1.00e + 05	1.03e + 05	3.92e + 04	**2.20e + 03**	1.97e + 09
	Worst	*6.46e + 05*	1.53e + 07	7.79e + 06	1.62e + 06	5.81e + 07	**4.43e + 04**	1.18e + 10
	Time(S)	*27.8839*	48.8842	**27.6130**	66.9520	31.2778	44.6840	31.7667
	Rank	2	4	5	3	6	1	7
F20	Mean	3.04e + 03	**2.76e + 03**	3.59e + 03	3.36e + 03	*2.95e + 03*	3.36e + 03	4.89e + 03
	Std	**2.17e + 02**	3.16e + 02	3.73e + 02	3.10e + 02	*2.64e + 02*	3.82e + 02	2.77e + 02
	Best	2.67e + 03	**2.13e + 03**	2.83e + 03	2.75e + 03	2.44e + 03	*2.32e + 03*	4.32e + 03
	Worst	3.69e + 03	**3.36e + 03**	4.20e + 03	3.85e + 03	*3.56e + 03*	4.05e + 03	5.39e + 03
	Time(S)	*9.6505*	29.0891	**9.5226**	24.3271	13.6634	15.0948	14.0242
	Rank	3	1	6	5	2	4	7
F21	Mean	2.75e + 03	**2.50e + 03**	2.89e + 03	2.80e + 03	2.87e + 03	*2.62e + 03*	3.23e + 03
	Std	*5.83e + 01*	**4.37e + 01**	1.21e + 02	6.90e + 01	7.19e + 01	7.22e + 01	8.21e + 01
	Best	2.62e + 03	**2.42e + 03**	2.65e + 03	2.64e + 03	2.69e + 03	*2.47e + 03*	3.04e + 03
	Worst	2.91e + 03	**2.63e + 03**	3.21e + 03	2.95e + 03	3.02e + 03	*2.81e + 03*	3.50e + 03
	Time(S)	**9.4044**	26.8629	*9.7493*	23.8923	13.5360	15.1159	13.1449
	Rank	3	1	6	4	5	2	7
F22	Mean	*9.62e + 03*	**8.43e + 03**	1.18e + 04	1.04e + 04	1.27e + 04	1.09e + 04	1.69e + 04
	Std	1.45e + 03	1.47e + 03	1.36e + 03	1.07e + 03	*9.20e + 02*	2.37e + 03	**7.68e + 02**
	Best	**2.30e + 03**	*3.21e + 03*	9.03e + 03	8.40e + 03	9.42e + 03	**2.30e + 03**	1.55e + 04
	Worst	*1.21e + 04*	**1.07e + 04**	1.47e + 04	1.39e + 04	1.51e + 04	1.32e + 04	1.84e + 04
	Time(S)	**11.6282**	29.1768	*11.8118*	29.3330	14.9062	18.1796	15.5930
	Rank	2	1	5	3	6	4	7
F23	Mean	3.42e + 03	*3.19e + 03*	3.58e + 03	3.60e + 03	3.97e + 03	**3.18e + 03**	4.61e + 03
	Std	**1.05e + 02**	*1.06e + 02*	1.43e + 02	1.56e + 02	2.20e + 02	1.17e + 02	2.97e + 02
	Best	3.13e + 03	*3.01e + 03*	3.26e + 03	3.29e + 03	3.58e + 03	**2.92e + 03**	4.04e + 03
	Worst	3.64e + 03	*3.46e + 03*	3.85e + 03	4.05e + 03	4.47e + 03	**3.39e + 03**	5.32e + 03
	Time(S)	**14.3041**	34.3205	*14.8105*	36.6722	18.9227	22.5017	18.9982
	Rank	3	2	4	5	6	1	7
F24	Mean	4.00e + 03	*3.41e + 03*	3.72e + 03	4.16e + 03	4.20e + 03	**3.26e + 03**	5.04e + 03
	Std	*1.54e + 02*	1.58e + 02	1.70e + 02	2.44e + 02	2.15e + 02	**1.15e + 02**	3.74e + 02
	Best	3.63e + 03	*3.13e + 03*	3.36e + 03	3.74e + 03	3.78e + 03	**3.08e + 03**	4.17e + 03
	Worst	4.38e + 03	*3.85e + 03*	4.09e + 03	4.71e + 03	4.75e + 03	**3.60e + 03**	5.92e + 03
	Time(S)	**12.6578**	31.9741	*13.0079*	31.4514	16.4495	20.7056	17.4785
	Rank	4	2	3	5	6	1	7
F25	Mean	*3.11e + 03*	3.25e + 03	3.14e + 03	3.11e + 03	1.08e + 04	**3.10e + 03**	1.70e + 04
	Std	**2.00e + 01**	2.98e + 02	4.26e + 01	*3.19e + 01*	1.70e + 03	3.41e + 01	1.91e + 03
	Best	3.06e + 03	*2.99e + 03*	3.05e + 03	3.05e + 03	7.35e + 03	**2.97e + 03**	1.25e + 04
	Worst	*3.16e + 03*	4.83e + 03	3.24e + 03	3.18e + 03	1.58e + 04	**3.15e + 03**	2.01e + 04
	Time(S)	**13.5669**	32.8977	*14.0638*	33.1838	18.0946	21.3640	17.8314
	Rank	2	5	4	3	6	1	7
F26	Mean	1.26e + 04	**6.74e + 03**	1.31e + 04	*9.05e + 03*	1.40e + 04	1.01e + 04	1.84e + 04
	Std	1.43e + 03	**9.07e + 02**	1.68e + 03	2.92e + 03	*1.18e + 03*	2.74e + 03	1.22e + 03
	Best	6.28e + 03	4.29e + 03	8.87e + 03	**2.93e + 03**	1.08e + 04	*3.38e + 03*	1.52e + 04
	Worst	1.53e + 04	**9.46e + 03**	1.72e + 04	*1.16e + 04*	1.61e + 04	1.34e + 04	2.03e + 04
	Time(S)	**15.2126**	34.3017	*16.5255*	40.0201	19.9385	25.0428	19.6167
	Rank	4	1	5	2	6	3	7
F27	Mean	4.65e + 03	**3.66e + 03**	4.26e + 03	3.88e + 03	5.33e + 03	*3.71e + 03*	7.34e + 03
	Std	3.55e + 02	*1.90e + 02*	4.34e + 02	2.18e + 02	6.16e + 02	**1.48e + 02**	8.45e + 02
	Best	4.02e + 03	**3.34e + 03**	3.57e + 03	3.55e + 03	4.45e + 03	*3.38e + 03*	5.53e + 03
	Worst	5.67e + 03	*4.19e + 03*	5.63e + 03	4.69e + 03	7.13e + 03	**4.12e + 03**	1.07e + 04
	Time(S)	**17.5726**	37.4702	*18.2153*	44.5548	20.8221	28.9288	21.8975
	Rank	5	1	4	3	6	2	7
F28	Mean	**3.33e + 03**	4.17e + 03	3.43e + 03	3.34e + 03	8.92e + 03	*3.34e + 03*	1.32e + 04
	Std	*3.23e + 01*	8.37e + 02	5.44e + 01	**3.09e + 01**	1.09e + 03	3.96e + 01	1.48e + 03
	Best	**3.26e + 03**	3.38e + 03	3.33e + 03	*3.27e + 03*	6.72e + 03	3.29e + 03	1.08e + 04
	Worst	**3.42e + 03**	6.77e + 03	3.57e + 03	3.49e + 03	1.15e + 04	*3.44e + 03*	1.75e + 04
	Time(S)	**15.0866**	32.7569	*15.2965*	36.8765	19.2617	24.1614	19.1116
	Rank	1	5	4	3	6	2	7
F29	Mean	6.62e + 03	**4.19e + 03**	7.01e + 03	5.26e + 03	7.84e + 03	*4.73e + 03*	2.30e + 05
	Std	6.26e + 02	4.54e + 02	8.24e + 02	*4.48e + 02*	1.38e + 03	**3.52e + 02**	6.66e + 05
	Best	5.33e + 03	**3.42e + 03**	5.07e + 03	4.47e + 03	5.02e + 03	*3.96e + 03*	1.66e + 04
	Worst	8.40e + 03	*6.11e + 03*	8.87e + 03	6.56e + 03	1.22e + 04	**5.50e + 03**	4.64e + 06
	Time(S)	**12.6896**	30.9491	13.3404	32.7556	16.6581	20.3604	16.7952
	Rank	4	1	5	3	6	2	7
F30	Mean	2.03e + 07	*1.03e + 07*	8.91e + 07	1.25e + 07	2.36e + 08	**2.25e + 06**	9.63e + 09
	Std	*1.74e + 06*	1.15e + 07	2.94e + 07	2.45e + 06	1.29e + 08	**9.30e + 05**	3.51e + 09
	Best	1.60e + 07	*1.08e + 06*	2.33e + 07	8.18e + 06	6.65e + 07	**1.01e + 06**	2.66e + 09
	Worst	2.42e + 07	6.23e + 07	1.87e + 08	*1.84e + 07*	6.51e + 08	**6.02e + 06**	1.80e + 10
	Time(S)	**30.3556**	49.6496	*31.1941*	75.8256	33.7729	50.1868	34.4177
	Rank	4	2	5	3	6	**1**	7
Overall	FMR	2.52 *	2.52 *	4.69	3.59	5.31	**2.38**	7.00
	F-Rank	*2*	*2*	4	3	5	**1**	6

*A more precise value is 2.51724137931.

In terms of computational efficiency, the execution time of each algorithm is reported^31^. POA frequently requires the least computational overhead. It records the best (fastest) execution times in several scenarios, including all tested functions except $\:{F}_{1},\:{F}_{4},\:{F}_{19},\:and\:{F}_{20}$. Even in functions where it does not hold the absolute top speed, POA consistently maintains the second-best execution time, trailing the fastest algorithms by only fractions of a second. In summary, POA shows accurate exploitation performance and superior exploration performance on the unimodal and multimodal functions, has strong local extremum avoidance on composite functions, and delivers high-quality solutions rapidly without prohibitive computational costs.

**Fig. 6:**
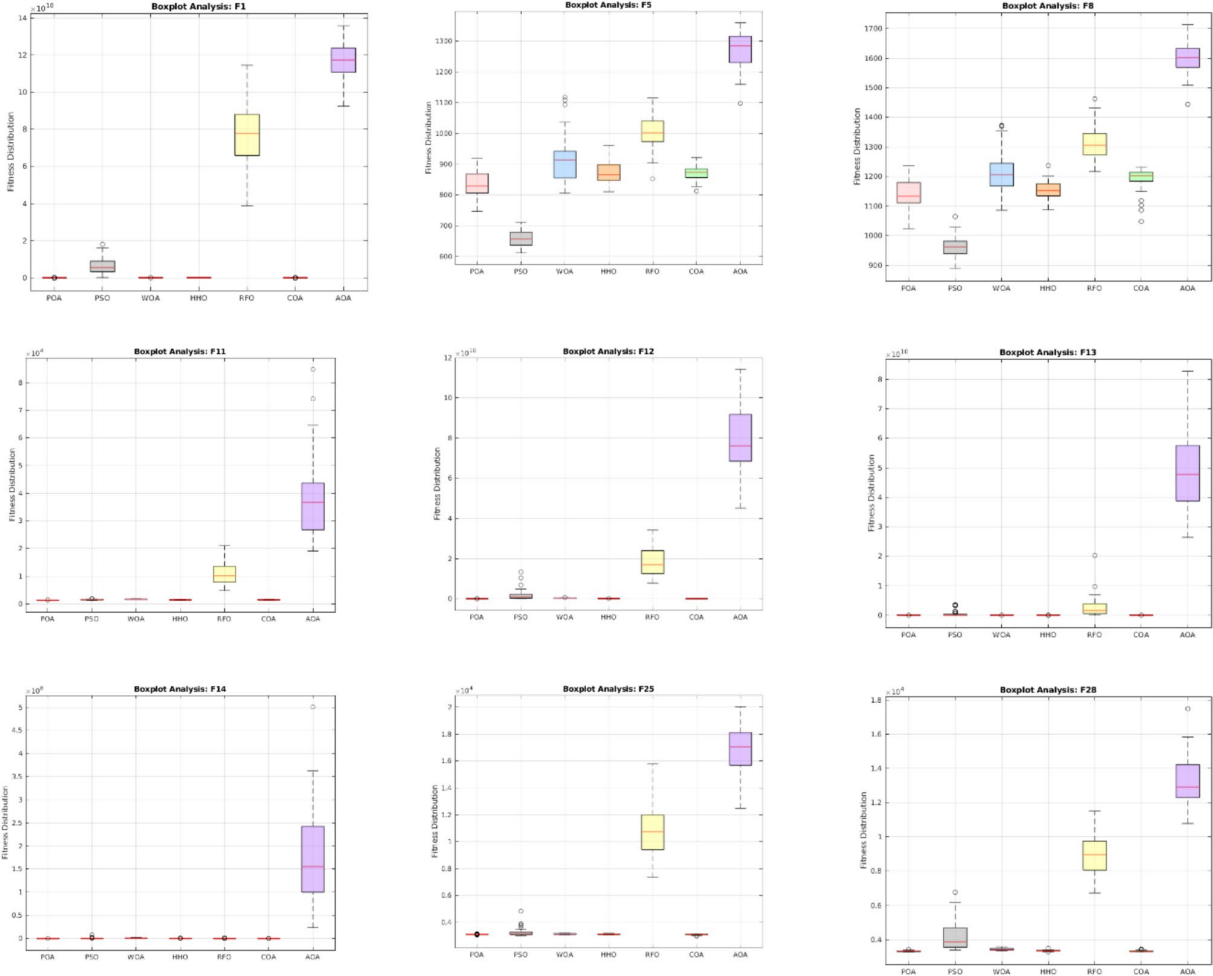
Boxplots of the different algorithms on the 50-dimensional CEC2017 functions.

To graphically illustrate the distribution of fitness values across the 51 independent runs, Fig. 6 presents boxplots for the evaluated algorithms on the 50-dimensional CEC2017 functions. In these visualizations, outlier data points (denoted by’$\:\circ\:$’) located above the upper whisker represent anomalously poor optimization runs. Conversely, outliers positioned below the lower whisker signify exceptionally high-quality solutions, indicating specific runs where the algorithm demonstrated highly effective exploration and exploitation.

As illustrated in Fig. 6, the distribution of the final optimization results for the POA is demonstrably superior to that of the six comparative algorithms. Notably, the POA exhibits a remarkably narrow data spread, indicating minimal variance across the independent runs compared to its competitors. This high consistency is particularly evident in functions $\:{F}_{1},\:{F}_{13},\:{F}_{25},\:and\:{F}_{28}$. These results confirm the algorithm’s exceptional stability and its ability to consistently locate high-quality solutions, even within highly complex search landscapes. Consequently, the boxplot analysis rigorously validates the overall effectiveness and strong robustness of the proposed POA.

### Convergence analysis

**Fig. 7:**
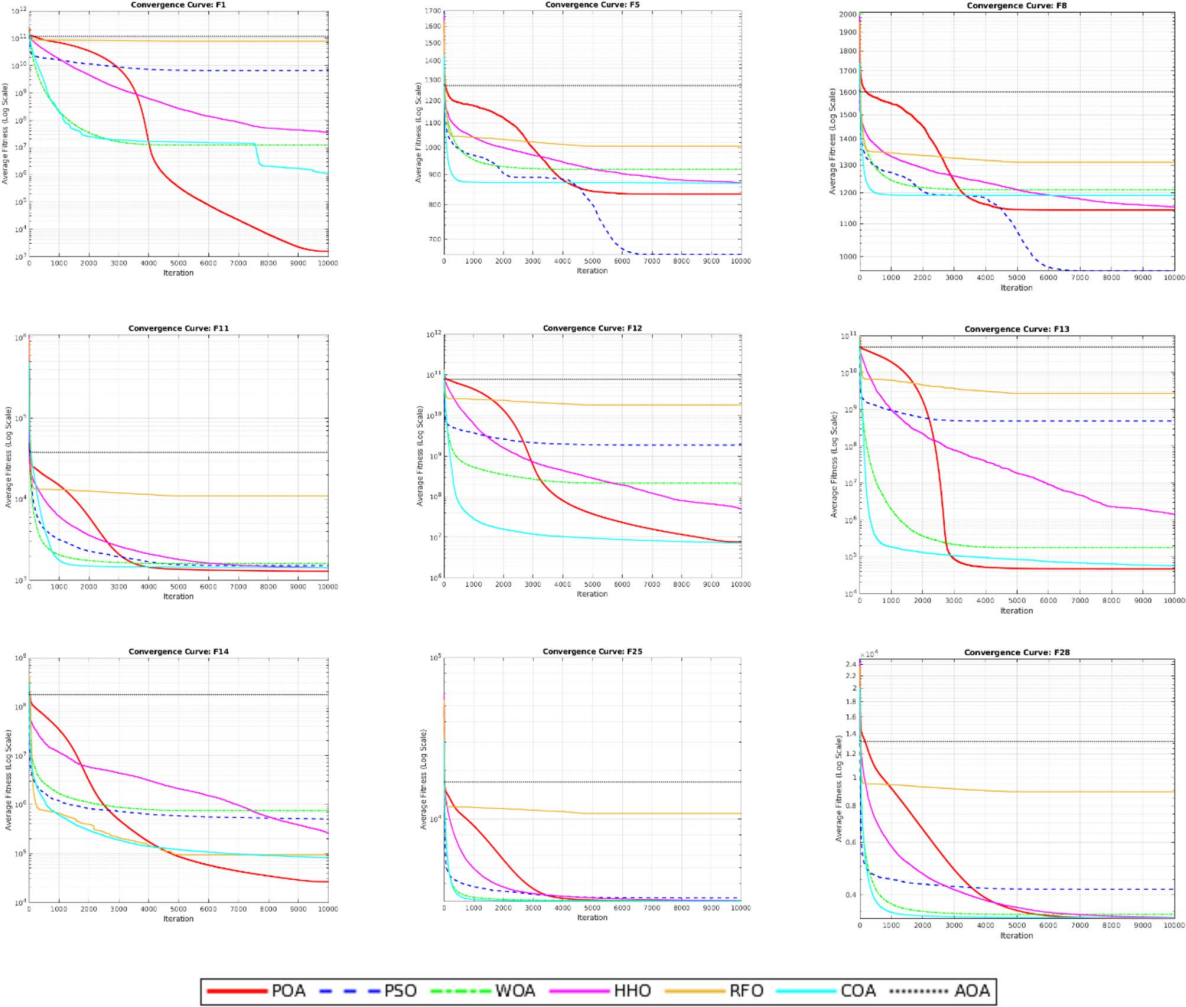
Convergence curves of the different algorithms on the 50-dimensional CEC2017 functions.

To evaluate the convergence dynamics and the balance between exploration and exploitation, a comparative convergence analysis was conducted between the proposed POA and six competitive algorithms. Figure 7 presents the convergence curves for these seven algorithms across the 50-dimensional CEC2017 benchmark suite. The selected test suite encompasses unimodal, multimodal, hybrid, and composition functions, providing a comprehensive assessment of the algorithms’ evolutionary trajectories and optimization behaviors throughout the iterative process.

As illustrated in Fig. 7, the convergence behaviors of the evaluated algorithms vary significantly across the diverse test functions. On the unimodal function $\:{F}_{1}$, the convergence curves demonstrate the superiority of the proposed POA, which exploits the search space to reach the optimal solution more effectively than its competitors. For the multimodal functions $\:{F}_{5}$ and $\:{F}_{8}$, the POA achieves superior results; while PSO exhibits competitive performance, the other algorithms prematurely converge to local optima during the early iterations.

Across the hybrid functions, the POA consistently displays the most robust global search performance. Specifically, on functions $\:{F}_{11},\:{F}_{12},\:{F}_{13},\:and\:{F}_{14}$, although the POA may not exhibit the fastest initial descent, it successfully escapes local optima as the iterations progress, ultimately converging near the global optimum. Conversely, the competing algorithms frequently stagnate at local optima without further improvement. Furthermore, the convergence curves for the highly complex composition functions (e.g., $\:{F}_{25}$ and $\:{F}_{28}$) reveal the POA’s strong local optima avoidance and high convergence accuracy. Ultimately, the performance on both hybrid and composition functions highlights the algorithm’s effective balance: executing broad global exploration during the initial phases, followed by precise, localized exploitation in the final stages. Overall, this convergence analysis substantiates the high efficacy and robustness of the proposed POA.

### Scalability analysis

Scalability analysis is a critical metric for evaluating an algorithm’s robustness across problem spaces of varying complexities. This section evaluates the optimization performance of the POA across multiple dimensionalities $\:(D\:=\:10,\:30,\:50,\:and\:100),$ with the empirical results detailed in Tables 6 and 7. As anticipated, the optimization accuracy experiences a general degradation as the problem dimension increases; this behavior is a standard manifestation of the exponentially expanding search space, commonly referred to as the curse of dimensionality.

**Table 6:** Mean and Std results of POA on different dimensions. (The best result is marked in bold, and underline indicate that the algorithm achieves suboptimal results.)

No.	Mean (10)	Mean (30)	Mean (50)	Mean (100)	Std (10)	Std (30)	Std (50)	Std (100)
$\:{F}_{1}$	*1.53e + 03*	**2.72e + 03**	**1.57e + 03**	**4.39e + 03**	**2.06e + 03**	**2.34e + 03**	**1.88e + 03**	**2.69e + 03**
$\:{F}_{3}$	**3.00e + 02**	5.15e + 04	4.03e + 04	2.86e + 05	*9.21e-10*	7.70e + 03	8.58e + 03	*1.98e + 04*
$\:{F}_{4}$	**4.00e + 02**	5.11e + 02	*5.75e + 02*	**7.71e + 02**	**2.86e-01**	**1.09e + 01**	**3.82e + 01**	**4.75e + 01**
$\:{F}_{5}$	5.37e + 02	*7.02e + 02*	*8.35e + 02*	*1.32e + 03*	1.03e + 01	3.29e + 01	3.87e + 01	7.02e + 01
$\:{F}_{6}$	6.14e + 02	6.49e + 02	6.57e + 02	6.66e + 02	5.34e + 00	*5.38e + 00*	*3.98e + 00*	*3.51e + 00*
$\:{F}_{7}$	7.50e + 02	1.14e + 03	*1.58e + 03*	*3.09e + 03*	1.53e + 01	7.99e + 01	1.27e + 02	2.21e + 02
$\:{F}_{8}$	8.20e + 02	*9.48e + 02*	*1.14e + 03*	*1.76e + 03*	*6.54e + 00*	2.74e + 01	4.70e + 01	1.00e + 02
$\:{F}_{9}$	9.99e + 02	*4.47e + 03*	*1.09e + 04*	**2.62e + 04**	8.89e + 01	*6.80e + 02*	**1.03e + 03**	*6.72e + 03*
$\:{F}_{10}$	1.55e + 03	*4.99e + 03*	*8.26e + 03*	*1.74e + 04*	2.35e + 02	7.70e + 02	1.15e + 03	3.15e + 03
$\:{F}_{11}$	1.17e + 03	**1.23e + 03**	**1.29e + 03**	1.71e + 04	3.86e + 01	**3.50e + 01**	**4.02e + 01**	3.62e + 03
$\:{F}_{12}$	3.38e + 04	*4.08e + 06*	**7.86e + 06**	**8.99e + 07**	*1.47e + 04*	*1.94e + 06*	**3.73e + 06**	**1.70e + 07**
$\:{F}_{13}$	7.94e + 03	*8.27e + 04*	**4.66e + 04**	**3.37e + 04**	5.20e + 03	*2.66e + 04*	**2.04e + 04**	**7.12e + 03**
$\:{F}_{14}$	1.51e + 03	3.59e + 04	**2.64e + 04**	**2.44e + 05**	1.76e + 02	2.04e + 04	**1.60e + 04**	**6.03e + 04**
$\:{F}_{15}$	1.80e + 03	2.93e + 04	*1.97e + 04*	**2.63e + 04**	2.68e + 02	1.47e + 04	*7.68e + 03*	**6.28e + 03**
$\:{F}_{16}$	1.74e + 03	2.98e + 03	3.79e + 03	7.35e + 03	1.13e + 02	4.19e + 02	5.24e + 02	8.99e + 02
$\:{F}_{17}$	1.75e + 03	2.44e + 03	4.00e + 03	6.36e + 03	**2.21e + 01**	2.57e + 02	*4.79e + 02*	*6.26e + 02*
$\:{F}_{18}$	5.34e + 03	*1.51e + 05*	*8.09e + 05*	**2.47e + 05**	*4.36e + 03*	*8.40e + 04*	**4.74e + 05**	**4.36e + 04**
$\:{F}_{19}$	3.15e + 03	9.08e + 05	*1.05e + 05*	*2.52e + 06*	1.96e + 03	2.20e + 05	*9.97e + 04*	*1.18e + 06*
$\:{F}_{20}$	2.04e + 03	2.42e + 03	3.04e + 03	*5.17e + 03*	**2.20e + 01**	**9.73e + 01**	**2.17e + 02**	5.92e + 02
$\:{F}_{21}$	**2.23e + 03**	2.51e + 03	2.75e + 03	3.73e + 03	5.42e + 01	**2.70e + 01**	*5.83e + 01*	*1.49e + 02*
$\:{F}_{22}$	**2.30e + 03**	*3.12e + 03*	*9.62e + 03*	*2.16e + 04*	2.24e + 01	1.77e + 03	1.45e + 03	3.11e + 03
$\:{F}_{23}$	2.63e + 03	3.01e + 03	3.42e + 03	4.80e + 03	4.78e + 01	7.52e + 01	**1.05e + 02**	5.53e + 02
$\:{F}_{24}$	**2.65e + 03**	3.39e + 03	4.00e + 03	6.07e + 03	1.43e + 02	1.14e + 02	*1.54e + 02*	**2.60e + 02**
$\:{F}_{25}$	2.93e + 03	2.92e + 03	*3.11e + 03*	**3.49e + 03**	*2.79e + 01*	2.26e + 01	**13.5669**	**4.20e + 01**
$\:{F}_{26}$	**2.90e + 03**	7.09e + 03	1.26e + 04	3.02e + 04	**1.71e + 02**	1.97e + 03	1.43e + 03	**3.00e + 03**
$\:{F}_{27}$	3.14e + 03	3.84e + 03	4.65e + 03	9.75e + 03	3.43e + 01	1.57e + 02	3.55e + 02	1.36e + 03
$\:{F}_{28}$	3.32e + 03	*3.22e + 03*	**3.33e + 03**	*3.62e + 03*	1.60e + 02	**1.53e + 01**	*3.23e + 01*	**4.72e + 01**
$\:{F}_{29}$	3.21e + 03	5.38e + 03	6.62e + 03	1.40e + 04	**2.89e + 01**	7.84e + 02	6.26e + 02	1.16e + 04
$\:{F}_{30}$	**4.42e + 04**	2.14e + 06	2.03e + 07	**2.18e + 07**	**2.18e + 05**	1.11e + 06	1.74e + 06	**6.75e + 06**

As detailed in Table 6, the proposed POA demonstrates exceptional performance at lower dimensions, yielding solutions that closely approximate the theoretical global optima. Notably, at $\:D\:=\:10,\:$for functions $\:{F}_{3}\:and\:{F}_{4},$ the mean fitness values achieved by the algorithm perfectly match the theoretical optima. Furthermore, the empirical data in Table 7 indicates that the POA maintains robust search capabilities at higher dimensions. Specifically, at $\:D\:=\:100,$ the algorithm achieves superior fitness values, securing the highest rank among all comparative algorithms. While this sustained high-dimensional performance may be partially attributed to the proportional scaling of the maximum number of function evaluations (MaxFEs), the algorithm’s ability to effectively leverage this expanded computational budget to dominate complex landscapes remains a highly significant finding.

**Fig. 8:**
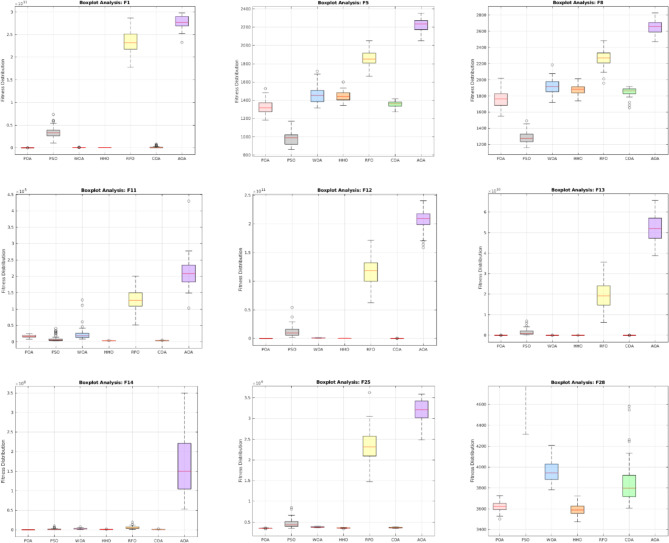
Boxplots of the different algorithms on the 100-dimensional CEC2017 functions.

As illustrated in Fig. 8, the boxplot distributions at $\:D\:=\:100$ demonstrate that the proposed POA retains robust local optima avoidance even within highly complex, high-dimensional search spaces. This sustained performance highlights the algorithm’s superior scalability and broader practical applicability compared to the competing algorithms.

**Table 7:** Optimization results for POA and its comparison algorithms on CEC2017 benchmark functions run independently 51, and the number of dimensions is 100.

NO	Metrics	POA	PSO	WOA	HHO	RFO	COA	AOA
F1	Mean	**4.39e + 03**	3.43e + 10	3.05e + 08	*2.66e + 08*	2.34e + 11	1.15e + 09	2.79e + 11
	Std	**2.69e + 03**	1.25e + 10	2.10e + 08	*2.88e + 07*	2.35e + 10	1.85e + 09	1.30e + 10
	Rank	1	5	3	2	6	4	7
F3	Mean	2.86e + 05	*1.70e + 05*	7.38e + 05	**8.72e + 04**	3.33e + 05	4.78e + 05	3.80e + 05
	Std	*1.98e + 04*	5.46e + 04	1.84e + 05	**1.53e + 04**	3.42e + 04	7.72e + 04	3.82e + 04
	Rank	3	2	7	1	4	6	5
F4	Mean	**7.71e + 02**	4.39e + 03	1.25e + 03	*8.94e + 02*	6.25e + 04	1.03e + 03	1.04e + 05
	Std	**4.75e + 01**	2.72e + 03	1.28e + 02	*6.55e + 01*	1.32e + 04	1.64e + 02	1.95e + 04
	Rank	1	5	4	2	6	3	7
F5	Mean	*1.32e + 03*	**9.82e + 02**	1.47e + 03	1.44e + 03	1.86e + 03	1.36e + 03	2.22e + 03
	Std	7.02e + 01	7.17e + 01	9.54e + 01	*5.27e + 01*	8.85e + 01	**3.12e + 01**	7.22e + 01
	Rank	2	1	5	4	6	3	7
F6	Mean	6.66e + 02	**6.20e + 02**	6.80e + 02	6.78e + 02	6.87e + 02	*6.62e + 02*	7.22e + 02
	Std	*3.51e + 00*	4.78e + 00	7.55e + 00	**3.45e + 00**	5.98e + 00	5.96e + 00	5.89e + 00
	Rank	3	1	5	4	6	2	7
F7	Mean	*3.09e + 03*	**1.59e + 03**	3.33e + 03	3.58e + 03	4.04e + 03	3.17e + 03	4.29e + 03
	Std	2.21e + 02	2.65e + 02	1.54e + 02	**1.36e + 02**	2.79e + 02	*1.56e + 02*	7.51e + 01
	Rank	2	1	4	5	6	3	7
F8	Mean	*1.76e + 03*	**1.29e + 03**	1.92e + 03	1.88e + 03	2.27e + 03	1.85e + 03	2.65e + 03
	Std	1.00e + 02	7.16e + 01	8.66e + 01	*6.34e + 01*	1.05e + 02	**5.55e + 01**	8.16e + 01
	Rank	2	1	5	4	6	3	7
F9	Mean	**2.62e + 04**	2.91e + 04	4.38e + 04	3.75e + 04	6.53e + 04	*2.84e + 04*	9.52e + 04
	Std	*6.72e + 03*	1.88e + 04	1.34e + 04	**5.48e + 03**	8.38e + 03	6.28e + 03	8.93e + 03
	Rank	1	3	5	4	6	2	7
F10	Mean	*1.74e + 04*	**1.47e + 04**	2.22e + 04	1.95e + 04	2.61e + 04	1.80e + 04	3.08e + 04
	Std	3.15e + 03	1.47e + 03	2.72e + 03	1.51e + 03	**1.22e + 03**	1.97e + 03	*1.33e + 03*
	Rank	2	1	5	4	6	3	7
F11	Mean	1.71e + 04	8.85e + 03	2.45e + 04	**3.17e + 03**	1.28e + 05	*3.45e + 03*	2.12e + 05
	Std	3.62e + 03	9.08e + 03	**2.20e + 04**	*2.77e + 02*	3.02e + 04	5.02e + 02	4.83e + 04
	Rank	4	3	5	1	6	2	7
F12	Mean	**8.99e + 07**	1.23e + 10	9.03e + 08	3.68e + 08	1.18e + 11	*1.12e + 08*	2.07e + 11
	Std	**1.70e + 07**	9.62e + 09	3.15e + 08	9.70e + 07	2.51e + 10	*6.38e + 07*	1.85e + 10
	Rank	1	5	4	3	6	2	7
F13	Mean	**3.37e + 04**	1.50e + 09	5.28e + 05	4.88e + 06	1.96e + 10	*1.07e + 05*	5.17e + 10
	Std	**7.12e + 03**	1.52e + 09	1.25e + 06	2.14e + 06	7.16e + 09	*9.44e + 04*	6.39e + 09
	Rank	1	5	3	4	6	2	7
F14	Mean	**2.44e + 05**	1.76e + 06	3.01e + 06	1.01e + 06	5.94e + 06	*8.22e + 05*	1.66e + 08
	Std	**6.03e + 04**	1.79e + 06	1.39e + 06	*2.84e + 05*	3.49e + 06	4.45e + 05	8.01e + 07
	Rank	1	4	5	3	6	2	7
F15	Mean	**2.63e + 04**	4.64e + 08	1.66e + 05	1.27e + 06	3.61e + 09	*4.95e + 04*	2.71e + 10
	Std	**6.28e + 03**	6.72e + 08	4.16e + 05	3.48e + 05	2.37e + 09	*2.07e + 04*	4.95e + 09
	Rank	1	5	3	4	6	2	7
F16	Mean	7.35e + 03	*6.37e + 03*	1.02e + 04	7.17e + 03	1.35e + 04	**6.03e + 03**	2.36e + 04
	Std	8.99e + 02	9.04e + 02	1.70e + 03	**6.29e + 02**	2.04e + 03	*8.24e + 02*	3.38e + 03
	Rank	4	2	5	3	6	1	7
F17	Mean	6.36e + 03	*5.90e + 03*	7.42e + 03	6.13e + 03	8.63e + 04	**5.59e + 03**	1.32e + 07
	Std	*6.26e + 02*	1.09e + 03	1.01e + 03	**6.24e + 02**	1.13e + 05	*6.26e + 02*	1.30e + 07
	Rank	4	2	5	3	6	1	7
F18	Mean	**2.47e + 05**	4.16e + 06	2.83e + 06	2.26e + 06	7.25e + 06	*1.56e + 06*	3.04e + 08
	Std	**4.36e + 04**	2.94e + 06	1.39e + 06	*9.86e + 05*	5.72e + 06	1.06e + 06	2.08e + 08
	Rank	1	5	4	3	6	2	7
F19	Mean	*2.52e + 06*	2.16e + 08	1.79e + 07	5.11e + 06	3.79e + 09	**6.25e + 04**	2.88e + 10
	Std	*1.18e + 06*	3.27e + 08	1.10e + 07	1.99e + 06	2.36e + 09	**4.00e + 04**	6.71e + 09
	Rank	2	5	4	3	6	1	7
F20	Mean	*5.17e + 03*	**4.86e + 03**	6.44e + 03	5.88e + 03	5.25e + 03	5.68e + 03	8.19e + 03
	Std	5.92e + 02	5.79e + 02	6.23e + 02	4.89e + 02	4.87e + 02	*4.77e + 02*	**3.72e + 02**
	Rank	2	1	6	5	3	4	7
F21	Mean	3.73e + 03	**3.07e + 03**	3.91e + 03	3.91e + 03	4.31e + 03	*3.44e + 03*	4.79e + 03
	Std	*1.49e + 02*	**1.12e + 02**	2.34e + 02	2.15e + 02	2.47e + 02	1.82e + 02	2.08e + 02
	Rank	3	1	4	5	6	2	7
F22	Mean	*2.16e + 04*	**1.70e + 04**	2.49e + 04	2.26e + 04	2.92e + 04	2.35e + 04	3.42e + 04
	Std	3.11e + 03	**1.21e + 03**	2.33e + 03	1.55e + 03	1.37e + 03	3.17e + 03	*1.29e + 03*
	Rank	2	1	5	3	6	4	7
F23	Mean	4.80e + 03	*4.43e + 03*	4.74e + 03	4.67e + 03	6.49e + 03	**4.04e + 03**	7.34e + 03
	Std	5.53e + 02	*2.30e + 02*	2.89e + 02	2.55e + 02	6.81e + 02	**1.93e + 02**	4.52e + 02
	Rank	5	2	4	3	6	1	7
F24	Mean	6.07e + 03	*5.64e + 03*	6.09e + 03	5.90e + 03	9.19e + 03	**4.81e + 03**	1.18e + 04
	Std	**2.60e + 02**	4.38e + 02	3.94e + 02	*3.19e + 02*	7.69e + 02	3.21e + 02	9.15e + 02
	Rank	4	2	5	3	6	1	7
F25	Mean	**3.49e + 03**	4.68e + 03	3.81e + 03	*3.57e + 03*	2.33e + 04	3.66e + 03	3.19e + 04
	Std	**4.20e + 01**	1.03e + 03	9.60e + 01	*6.86e + 01*	3.59e + 03	9.67e + 01	2.85e + 03
	Rank	1	5	4	2	6	3	7
F26	Mean	3.02e + 04	**1.85e + 04**	3.19e + 04	*2.39e + 04*	4.45e + 04	2.61e + 04	5.64e + 04
	Std	**3.00e + 03**	*3.03e + 03*	3.89e + 03	4.40e + 03	4.95e + 03	3.45e + 03	4.72e + 03
	Rank	4	1	5	2	6	3	7
F27	Mean	9.75e + 03	*4.10e + 03*	5.00e + 03	4.24e + 03	9.79e + 03	**4.08e + 03**	1.35e + 04
	Std	1.36e + 03	3.33e + 02	6.57e + 02	*3.12e + 02*	1.37e + 03	**2.06e + 02**	1.49e + 03
	Rank	5	2	4	3	6	1	7
F28	Mean	*3.62e + 03*	9.14e + 03	3.96e + 03	**3.59e + 03**	2.81e + 04	3.85e + 03	3.69e + 04
	Std	**4.72e + 01**	3.27e + 03	1.02e + 02	*5.27e + 01*	3.72e + 03	2.09e + 02	3.74e + 03
	Rank	2	5	4	1	6	3	7
F29	Mean	1.40e + 04	**7.12e + 03**	*1.42e + 04*	8.97e + 03	2.41e + 04	8.40e + 03	1.28e + 06
	Std	1.45e + 03	**7.52e + 02**	1.76e + 03	*7.57e + 02*	1.47e + 04	6.87e + 02	1.13e + 06
	Rank	4	1	5	3	6	2	7
F30	Mean	**2.18e + 07**	1.21e + 09	2.72e + 08	*2.57e + 07*	1.24e + 10	2.60e + 07	4.33e + 10
	Std	**6.75e + 06**	1.54e + 09	1.17e + 08	*6.88e + 06*	7.81e + 09	1.57e + 08	7.78e + 09
	Rank	1	5	4	2	6	3	7
Overall	FMR	**2.38**	2.83	4.52	3.07	5.83	*2.45*	6.93
	F-Rank	**1**	3	5	4	6	*2*	7

### Statistical tests

Due to the inherent stochasticity of metaheuristic algorithms, comparing mean fitness values alone is insufficient to definitively establish algorithmic superiority. Therefore, to rigorously validate the performance of the POA, non-parametric statistical tests were conducted using the optimization results from the 50-dimensional CEC 2017 functions. Specifically, a Wilcoxon rank-sum test was performed at a $\:5\%$ significance level. The POA was designated as the control algorithm and evaluated pairwise against each comparative algorithm to compute the respective *p*-values, which are summarized in Table 8. In this evaluation, the symbols $\:{\prime\:}+{\prime\:}$ and $\:{\prime\:}-{\prime\:}$ denote whether the POA exhibits a statistically significant advantage or is significantly outperformed by the competitor, respectively. Out of the *\:174* pairwise comparisons, the POA demonstrated significant superiority in *\:162* instances. These results provide robust statistical evidence that the POA consistently outperforms the competing algorithms. Furthermore, the Friedman test as a non-parametric, rank-based statistical method was employed to determine whether significant performance differences exist across multiple algorithm distributions simultaneously. While pairwise tests evaluate specific matchups, the Friedman test provides a macroscopic evaluation of overall algorithmic superiority. Accordingly, this test was utilized to assess the comprehensive optimization capabilities of the POA across the 50 and 100-dimensional CEC2017 benchmark functions. As detailed in Tables 5 and 7, the POA achieved the first-place ranking among all evaluated algorithms. These overarching statistical results rigorously validate the efficacy of the POA balanced exploration and exploitation strategies.

**Table 8:** Wilcoxon rank-sum test results of the different algorithms on the 50-dimensional CEC2017 functions.

Fun.	POA vs. PSO	POA vs. WOA	POA vs. HHO	POA vs. RFO	POA vs. COA	POA vs. AOA
$\:{F}_{1}$	3.30e-18 (+)	3.30e-18 (+)	3.30e-18 (+)	3.30e-18 (+)	3.30e-18 (+)	3.30e-18 (+)
$\:{F}_{3}$	2.23e-10 (+)	3.75e-14 (+)	3.30e-18 (+)	3.30e-18 (+)	3.15e-11 (+)	3.30e-18 (+)
$\:{F}_{4}$	9.38e-17 (+)	1.21e-13 (+)	5.71e-03 (+)	3.30e-18 (+)	8.39e-04 (+)	3.30e-18 (+)
$\:{F}_{5}$	3.30e-18 (+)	5.74e-10 (+)	7.80e-06 (+)	2.68e-17 (+)	4.41e-06 (+)	3.30e-18 (+)
$\:{F}_{6}$	3.30e-18 (+)	2.26e-17 (+)	5.89e-16 (+)	1.60e-07 (+)	6.68e-01 (-)	3.30e-18 (+)
$\:{F}_{7}$	3.30e-18 (+)	2.86e-04 (+)	4.26e-10 (+)	7.21e-12 (+)	4.54e-03 (+)	2.58e-18 (+)
$\:{F}_{8}$	3.94e-18 (+)	2.06e-07 (+)	9.17e-02 (-)	5.61e-18 (+)	3.52e-07 (+)	3.30e-18 (+)
$\:{F}_{9}$	3.50e-18 (+)	4.70e-18 (+)	4.18e-18 (+)	3.30e-18 (+)	2.70e-15 (+)	3.30e-18 (+)
$\:{F}_{10}$	7.66e-09 (+)	3.57e-09 (+)	6.39e-01 (-)	5.00e-16 (+)	2.29e-11 (+)	3.30e-18 (+)
$\:{F}_{11}$	1.32e-16 (+)	3.30e-18 (+)	1.81e-14 (+)	3.30e-18 (+)	7.34e-16 (+)	3.30e-18 (+)
$\:{F}_{12}$	3.39e-16 (+)	3.30e-18 (+)	4.99e-18 (+)	3.30e-18 (+)	6.25e-01 (-)	3.30e-18 (+)
$\:{F}_{13}$	2.11e-12 (+)	3.04e-16 (+)	3.30e-18 (+)	3.30e-18 (+)	3.06e-02 (+)	3.30e-18 (+)
$\:{F}_{14}$	1.91e-13 (+)	3.72e-18 (+)	2.26e-17 (+)	9.79e-01 (-)	1.18e-08 (+)	3.30e-18 (+)
$\:{F}_{15}$	9.23e-04 (+)	1.72e-14 (+)	3.72e-18 (+)	9.34e-09 (+)	4.91e-01 (-)	3.30e-18 (+)
$\:{F}_{16}$	1.07e-12 (+)	3.83e-13 (+)	3.52e-04 (+)	4.95e-12 (+)	7.42e-11 (+)	3.30e-18 (+)
$\:{F}_{17}$	1.05e-16 (+)	8.62e-01 (-)	2.48e-05 (+)	3.02e-10 (+)	7.05e-15 (+)	3.30e-18 (+)
$\:{F}_{18}$	8.25e-01 (-)	7.48e-17 (+)	2.58e-09 (+)	2.75e-07 (+)	1.79e-01 (-)	3.30e-18 (+)
$\:{F}_{19}$	4.22e-01 (-)	3.56e-12 (+)	6.59e-14 (+)	2.56e-15 (+)	5.95e-14 (+)	3.30e-18 (+)
$\:{F}_{20}$	9.60e-08 (+)	1.17e-09 (+)	1.40e-05 (+)	9.83e-02 (-)	2.86e-04 (+)	3.72e-18 (+)
$\:{F}_{21}$	3.30e-18 (+)	4.10e-12 (+)	4.20e-05 (+)	1.27e-13 (+)	1.21e-10 (+)	3.30e-18 (+)
$\:{F}_{22}$	2.19e-09 (+)	3.29e-09 (+)	8.54e-05 (+)	1.47e-16 (+)	8.04e-06 (+)	3.30e-18 (+)
$\:{F}_{23}$	3.15e-10 (+)	3.43e-10 (+)	2.43e-10 (+)	7.96e-18 (+)	3.17e-15 (+)	3.30e-18 (+)
$\:{F}_{24}$	3.30e-18 (+)	6.26e-13 (+)	3.03e-03 (+)	7.07e-09 (+)	3.30e-18 (+)	3.30e-18 (+)
$\:{F}_{25}$	9.05e-03 (+)	3.75e-06 (+)	1.66e-03 (+)	3.30e-18 (+)	2.41e-02 (+)	3.30e-18 (+)
$\:{F}_{26}$	1.84e-16 (+)	4.74e-01 (-)	1.43e-12 (+)	6.15e-08 (+)	9.68e-11 (+)	3.50e-18 (+)
$\:{F}_{27}$	3.30e-18 (+)	1.82e-06 (+)	3.39e-16 (+)	1.56e-08 (+)	3.30e-18 (+)	3.30e-18 (+)
$\:{F}_{28}$	4.01e-17 (+)	6.23e-16 (+)	3.74e-05 (+)	3.30e-18 (+)	4.30e-01 (-)	3.30e-18 (+)
$\:{F}_{29}$	3.30e-18(+)	1.52e-01(-)	1.27e-17(+)	2.95e-07(+)	3.30e-18(+)	3.30e-18(+)
$\:{F}_{30}$	1.47e-14(+)	3.30e-18(+)	5.97e-17(+)	3.30e-18(+)	3.30e-18(+)	3.30e-18(+)

## POA for engineering design problems

Real-world engineering problems are inherently more complex than standard mathematical benchmarks due to the presence of numerous physical constraints and highly coupled decision variables. While the CEC2017 benchmark functions effectively simulate the diverse search spaces of practical optimization problems, it remains essential to evaluate the algorithm in applied scenarios. To further validate its practical applicability, this section applies the POA to four classic engineering design problems: the three-bar truss design, the gear train design, the tension/compression spring design, and the corrugated bulkhead design. For these experiments, the population size was set to 50, with a maximum of 1,000 iterations. The algorithm was executed for 25 independent runs, and the best-obtained results were compared against previous algorithms.

### Three-bar truss design problem

**Fig. 9:**
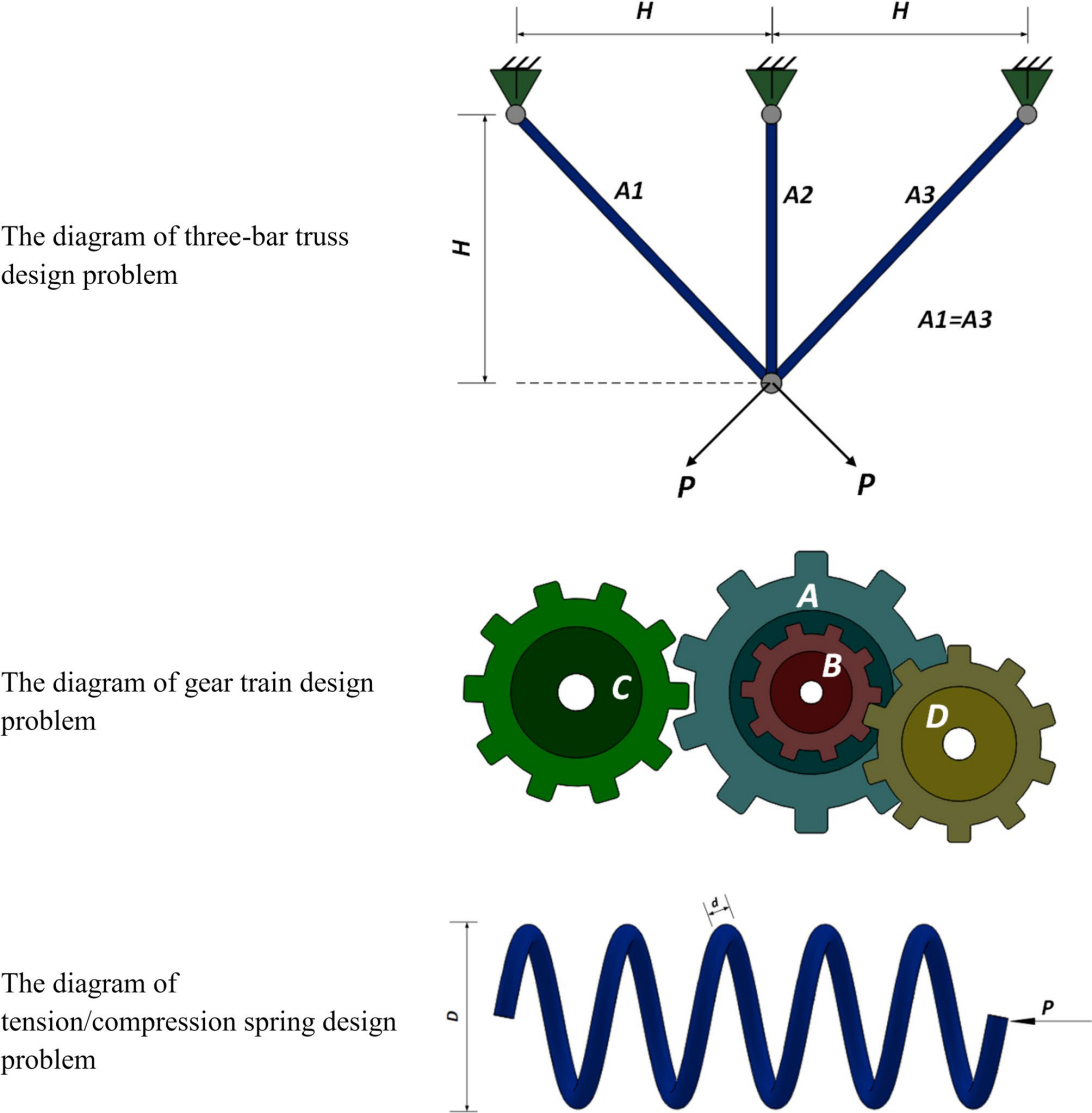
Structural diagram of engineering problems.

The objective of the three-bar truss design problem^32^, illustrated in Fig. 9, is to minimize the total volume of the structure. This is achieved by optimizing two continuous decision variables, $\:{A}_{1}\:$and $\:{A}_{2}\:$, which represent the cross-sectional areas of the truss members. The optimization process is subject to specific stress constraints applied to each individual member. The complete mathematical formulation for this problem is provided in Eq. (17).

Consider:

$$\:X=\left[{X}_{1},\:{X}_{2}\right]=\:\left[{A}_{1},\:{A}_{2}\right]$$

Minimize:

$$\:f\left(X\right)=\:\left(2\sqrt{2}{X}_{1}+{X}_{2}\right)\times\:H$$

Subject to:

$$\:{g}_{1}\left(X\right)=\:\frac{\sqrt{2}{X}_{1}+{X}_{2}}{\sqrt{2}{X}_{1}^{2}+{2X}_{1}{X}_{2}}P-\sigma\:\:\le\:0,$$

17 $$\:{g}_{2}\left(X\right)=\:\frac{{X}_{2}}{\sqrt{2}{X}_{1}^{2}+{2X}_{1}{X}_{2}}P-\sigma\:\:\le\:0,$$

$$\:{g}_{3}\left(X\right)=\:\frac{1}{\sqrt{2}{X}_{2}+{X}_{1}}P-\sigma\:\:\le\:0,$$

$$\:H=100\:cm,\:P=2kN/{cm}^{3},\:\sigma\:=2\:kN/{cm}^{3}.$$.

Variable range:

$$\:{0\le\:X}_{1},\:{X}_{2}\le\:1.$$

The comparative results for the three-bar truss design problem are summarized in Table 9. The data indicates that the POA achieves the optimal fitness value, performing consistently alongside PSO and RFO. Regarding computational efficiency, while the WOA requires less execution time than the proposed algorithm, its optimization accuracy remains unsatisfactory. This suggests that the POA provides a superior balance between search efficacy and computational overhead, effectively outperforming the other methods in locating the global optimum.

### Gear train design problem

the gear train design problem seeks to optimize the gear ratio through the selection of suitable tooth numbers for a compound gear system^33^. schematically illustrated in Fig. 9, This problem is characterized by four integer decision variables representing the tooth counts of gears $\:A,\:B,\:C,\:$and *\:D* $\:({n}_{A},\:{n}_{B},\:{n}_{C},\:{n}_{D})$. The objective function and associated design constraints are mathematically expressed in Eq. (18), requiring the algorithm to navigate a discrete search space to optimize the gear ratio.

Consider:

$$\:X=\left[{X}_{1},\:{X}_{2},\:{X}_{3},\:{X}_{4}\right]=\:\left[{n}_{A},\:{n}_{B},\:{n}_{C},\:{n}_{D}\right]$$

Minimize:

18 $$\:f\left(X\right)={\left(\frac{1}{6.931}-\frac{{X}_{2}\:{X}_{3}}{{X}_{1}{X}_{4}}\right)}^{2}$$

Variable range:

$$\:{X}_{1},\:{X}_{2},\:{X}_{3},\:{X}_{4}\:\in\:\{12,\:13,\:14,\:\dots\:,\:60\}$$

Table 10 presents the optimal design configurations achieved by the POA and its competitors, The data reveals that while several algorithms achieved the same minimum fitness value as the POA, others _specifically the AOA _ exhibited relatively poor performance. In terms of computational efficiency, both the POA and WOA recorded significantly shorter execution times than the other methods. However, the POA demonstrated superior overall performance by securing a higher degree of optimization accuracy with even lower computational overhead than the WOA.

### Tension/compression spring design problem

The third application is the tension/compression spring design problem, illustrated in Fig. 9. The primary objective of this optimization challenge is to minimize the total weight of the spring. The design space is governed by three continuous decision variables: the wire diameter $\:\left(d\right)$, the mean coil diameter $\:\left(D\right)$, and the number of active coils $\:\left(N\right)$. Several constraints must be satisfied, including limits on shear stress, surge frequency, and minimum deflection. The mathematical formulation of the objective function and its associated constraints is presented in Eq. (19)

Consider:

$$\:X=\left[{X}_{1},\:{X}_{2},\:{X}_{3}\right]=\:\left[d,\:D,\:N\right]$$

Minimize:

$$\:f\left(X\right)=({X}_{3}+2){X}_{2}{X}_{1}^{2}$$

Subject to:

$$\:{g}_{1}\left(X\right)=1-\frac{{X}_{2}^{3}{X}_{3}}{71785{X}_{1}^{4}}\le\:0,$$

19 $$\:{g}_{2}\left(X\right)=\frac{4{X}_{2}^{2}-{X}_{1}{X}_{2}}{12566\left({{X}_{2}X}_{1}^{3}-{X}_{1}^{4}\right)}+\frac{1}{5108{X}_{1}^{2}}-1\le\:0,$$

$$\:{g}_{3}\left(X\right)=1-\frac{140.45{X}_{1}}{{X}_{2}^{2}{X}_{3}}\le\:0,$$

$$\:{g}_{4}\left(X\right)=\frac{{X}_{1}+{X}_{2}}{1.5}-1\le\:0.$$.

Variables range:

$$\:0.05\le\:{X}_{1}\le\:2.0,\:0.25\le\:{X}_{2}\le\:1.3,\:and\:2\le\:{X}_{3}\le\:15.0.$$

Table 11 presents a comparative summary of the optimal design configurations achieved by the POA and its competitors, utilizing data from 25 independent runs alongside reported values from the literature. The results indicate that the POA successfully converged to the best-known fitness value, matching the peak performance of PSO, WOA, RFO and COA, in contrast, algorithms such as HHO and AOA exhibited relatively poor optimization accuracy for the tension/compression spring design problem. Overall, the POA demonstrates superior optimization efficacy, providing high-quality solutions with a notable advantage in computational efficiency compared to the alternative methods.

## POA for trajectory planning

Trajectory planning is a fundamental challenge in robotic research, particularly for high-degree-of-freedom (DOF) systems. It involves the precise calculation of a sequence of waypoints, including the position, orientation, velocity, and acceleration of the end-effector as it moves from a starting configuration to a target pose. Rather than moving linearly between points, the manipulator must follow a coordinated path that accounts for varying joint speeds, distinct axes of motion, and environmental obstacles. The primary objective is to ensure smooth, controlled, and vibration-free motion that avoids collisions while optimizing the dynamic profile. As the system’s degrees of freedom increase, identifying these intermediate waypoints becomes significantly more complex, as the algorithm must harmonize acceleration and distance parameters across all joints to reach the desired target effectively^20^.

**Table 9:** Best results of various methods for three-bar truss design problem.

Metric	POA	PSO	WOA	HHO	RFO	COA	AOA
$\:{x}_{1}$	0.78869	0.78868	0.78883	0.78815	0.78868	0.78869	0.78206
$\:{x}_{2}$	0.40822	0.40823	0.40780	0.40973	0.40825	0.40822	0.42888
$\:{g}_{1}$	3.11e-15	-2.66e-15	-3.03e-10	0.00e + 00	-4.02e-14	-9.04e-08	-1.16e-03
$\:{g}_{2}$	-1.46e + 00	-1.46e + 00	1.46e + 00	-1.46e + 00	-1.46e + 00	-1.46e + 00	-1.44e + 00
$\:{g}_{3}$	-5.36e-01	-5.36e-01	-5.35e-01	-5.38e-01	-5.36e-01	-5.36e-01	-5.60e-01
Time	0.86849	2.38010	**0.74590**	1.82649	1.09308	1.13952	0.77975
*\:f*	**263.89584**	**263.89584**	263.89586	263.89604	**263.89584**	263.89586	264.08674

**Table 10:** Best results of various methods for gear train design problem.

Metric	POA	PSO	WOA	HHO	RFO	COA	AOA
$\:{x}_{1}$	48.91864	49.11908	42.58686	43.25736	48.73203	43.23030	54.04355
$\:{x}_{2}$	15.98584	18.50664	19.15833	19.48565	18.86894	18.65105	36.60435
$\:{x}_{3}$	18.96863	16.36616	15.69282	15.72074	15.75141	15.92160	12.10216
$\:{x}_{4}$	43.20990	42.78257	48.93269	49.23272	43.15119	48.78226	57.09833
$\:{g}_{1}$	0.00e + 00	0.00e + 00	0.00e + 00	0.00e + 00	0.00e + 00	0.00e + 00	0.00e + 00
Time	**0.44035**	2.04074	0.44982	1.23245	0.75094	0.64008	0.52649
*\:f*	2.7009e-12*	2.7009e-12*	2.7009e-12*	2.7009e-12*	2.7009e-12*	2.7009e-12*	8.8876e-10^#^

* A more precise value is 2.700857148886513e-12; ^#^ A more precise value is 8.887614372714457e-10.

**Table 11:** Best results of various methods for tension compression spring design.

Metric	POA	PSO	WOA	HHO	RFO	COA	AOA
$\:{x}_{1}$	0.05142	0.05170	0.05186	0.05322	0.05169	0.05143	0.05000
$\:{x}_{2}$	0.35034	0.35691	0.36081	0.39461	0.35671	0.35045	0.31043
$\:{x}_{3}$	11.67798	11.27771	11.05321	9.37001	11.28937	11.66893	15.00000
$\:{g}_{1}$	-4.33e-04	-4.44e-15	-7.84e-08	-6.66e-16	-1.04e-08	-1.94e-04	-1.13e-04
$\:{g}_{2}$	-6.98e-07	-6.66e-16	-7.41e-09	0.00e + 00	-1.06e-08	-1.87e-05	-1.73e-02
$\:{g}_{3}$	-4.04e + 00	-4.05e + 00	-4.06e + 00	-4.12e + 00	-4.05e + 00	-4.04e + 00	-3.86e + 00
$\:{g}_{4}$	-7.32e-01	-7.28e-01	-7.25e-01	-7.01e-01	-7.28e-01	-7.32e-01	-7.60e-01
Time	**0.73471**	2.40637	0.76782	1.97282	1.14718	1.19694	0.79451
*\:f*	**0.01267**	**0.01267**	**0.01267**	0.01271	**0.01267**	**0.01267**	0.01319

In this section, we will use the POA algorithm to find the optimal time of trajectory planning of a six-degree-of-freedom industrial robotic arm, taking into account the robot’s physical constraints. We have a predefined path with a starting point, an ending point, and three via points. Therefore, an eighth-degree polynomial was used to represent the positions, velocities, and accelerations of all the robot’s joints at any instant along the path. The results were compared with five state-of-the-art metaheuristic optimization algorithms.

### Robotic arm model

**Fig. 10:**
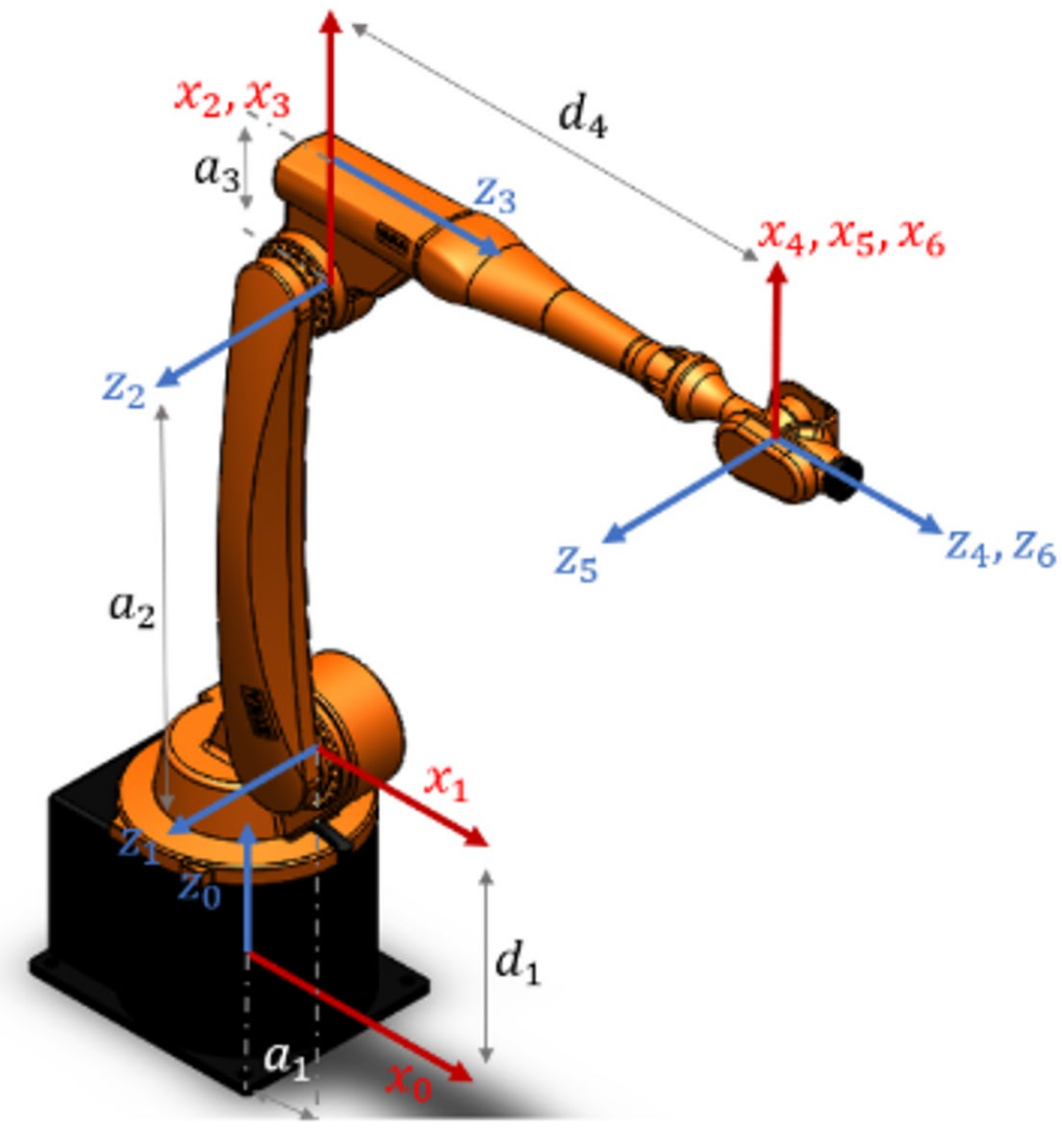
DH coordinate frames assignment for the KR20 R2810 manipulator.

The robot arm model used in this paper is the KR20-R1810 robot arm from KUKA, as shown in Fig. 10. The maximum reach range of the arm is 1813 mm, the motion rang and the maximum movement speed with rated payload of each joint is shown in Table 12. The arm is designed to work in the same way as the robot arm. Modeled using DH method, the parameters of each linkage and joint of KR20-R1810 robotic arm are shown in Table 13. In the Denavit Hartenberg method, robot kinematics is created using four main variables as follow.

i. link length $\:\left({a}_{i}\right)$: distance along $\:{x}_{i}$ from the intersection of the $\:{x}_{i}$ and $\:{z}_{i-1}$ axes to $\:{o}_{i}$.
ii. link twist $\:\left({\alpha\:}_{i}\right)$: the angle from $\:{z}_{i-1}$ to $\:{z}_{i}$ measured about $\:{x}_{i}$.
iii. joint offset $\:\left({d}_{i}\right)$: distance along $\:{z}_{i-1}$ from $\:{o}_{i-1}$ to the intersection of the $\:{x}_{i}$ and $\:{z}_{i-1}$ axes.
iv. joint angle $\:\left({\theta\:}_{i}\right)$: the angle from $\:{x}_{i-1}$ to $\:{x}_{i}$ measured about $\:{z}_{i-1}$.

### Trajectory planning and polynomial function

Robotic arm trajectory planning constitutes a complex, high-dimensional, and non-linear optimization problem typically aimed at minimizing three primary objective functions: execution time, energy consumption, and jerk. In this study, the primary goal of the POA is to determine the optimal time allocation across the entire path. To achieve this, the overall trajectory is discretized into four distinct, continuous segments defined by an initial position, three intermediate via-points, and a final target destination. To guarantee higher-order kinematic continuity and smooth motion, each of these four segments is mathematically interpolated utilizing an eighth-order polynomial function.

**Table 12:** Motion rang and the maximum movement speed with rated payload of each joint of the robot.

Joint	Motion range	Maximum speed
1	$\:\pm\:180^\circ\:$	$\:200^\circ\:/S$
2	$\:-95^\circ\:/155^\circ\:$	$\:175^\circ\:/S$
3	$\:-138^\circ\:/175^\circ\:$	$\:190^\circ\:/S$
4	$\:\pm\:350^\circ\:$	$\:430^\circ\:/S$
5	$\:\pm\:130^\circ\:$	$\:430^\circ\:/S$
6	$\:\pm\:350^\circ\:$	$\:630^\circ\:/S$

The trajectory planning problem was formulated as a single-objective, constrained optimization problem is provided in Eq. (20).

$$\:X=\left[{X}_{1},\:{X}_{2},\:{X}_{3},{X}_{4}\right]=\:\left[{t}_{1},\:{t}_{2},\:{t}_{3},{t}_{4}\right]$$

Minimize:

$$\:f\left(X\right)=\:\sum\:_{i=1}^{4}d{X}_{i}+\lambda\:({P}_{v}\left(j\right)+{P}_{\theta\:lower}\left(j\right)+{P}_{\theta\:upper}\left(j\right))$$

Subject to:

$$\:{P}_{v}\left(j\right)=\mathrm{m}\mathrm{a}\mathrm{x}(0,\:{max}_{t}\left|{v}_{j}\left(t\right)\right|-{v}_{max,j})$$

$$\:{v}_{max,j}=\left[120,\:120,\:120,\:210,\:210,\:300\right],\:j=\mathrm{1,2},\dots\:,6$$

$$\:{P}_{\theta\:lower}\left(j\right)=\mathrm{m}\mathrm{a}\mathrm{x}(0,\:{\theta\:}_{min,j}-{min}_{t}{\theta\:}_{j}\left(t\right))$$

$$\:{\theta\:}_{min,j}=\left[-185,\:-185,\:-138,-350,-130,-350\right],\:j=\mathrm{1,2},\dots\:,6\:\:\:\:\:\:\:\:\:\:\:\:\:\:\:\:\:\:\:\:\:\:\:\:\:\:\:\:\:\:\:\:\:\:\:\:\:\:\:\:\:\:\left(20\right)$$

$$\:{P}_{\theta\:upper}\left(j\right)=\mathrm{m}\mathrm{a}\mathrm{x}(0,{max}_{t}{\theta\:}_{j}\left(t\right)-{\theta\:}_{max,j})$$

$$\:{\theta\:}_{max,j}=\left[185,\:65,\:175,\:350,\:130,\:350\right],\:j=\mathrm{1,2},\dots\:,6$$

Variables range:

$$\:0.1\le\:{X}_{i}\le\:5.0\:Sec$$

Where $\:{P}_{v}\left(j\right)$ velocity penalty, $\:{P}_{\theta\:lower}\left(j\right)$ is lower joint angle penalty, $\:{P}_{\theta\:upper}\left(j\right)$ is upper joint angle penalty, $\:{v}_{max,j}$ is maximum angle velocity limits, $\:{\theta\:}_{min,j}$ is minimum angle limits, $\:{\theta\:}_{max,j}$ is maximum angle limits, $\:\lambda\:=1000$ is the penalty weight.

**Table 13:** DH parameters for the KR20 R2810 manipulator.

Link	$\:{a}_{i}$	$\:{\alpha\:}_{i}$	$\:{d}_{i}$	$\:{\theta\:}_{i}$
1	$\:{a}_{1}=160$	*\:90*	$\:{d}_{1}=520$	$\:{\theta\:}_{1}$
2	$\:{a}_{2}=780$	*\:0*	*\:0*	$\:{\theta\:}_{2}=90^\circ\:$
3	$\:{a}_{3}=150$	*\:90*	*\:0*	$\:{\theta\:}_{3}$
4	*\:0*	*\:-90*	$\:{d}_{4}=860$	$\:{\theta\:}_{4}$
5	*\:0*	*\:90*	*\:0*	$\:{\theta\:}_{5}$
6	*\:0*	*\:0*	*\:0*	$\:{\theta\:}_{6}$

Whereas. Dimensions mm.

**Fig. 11:**
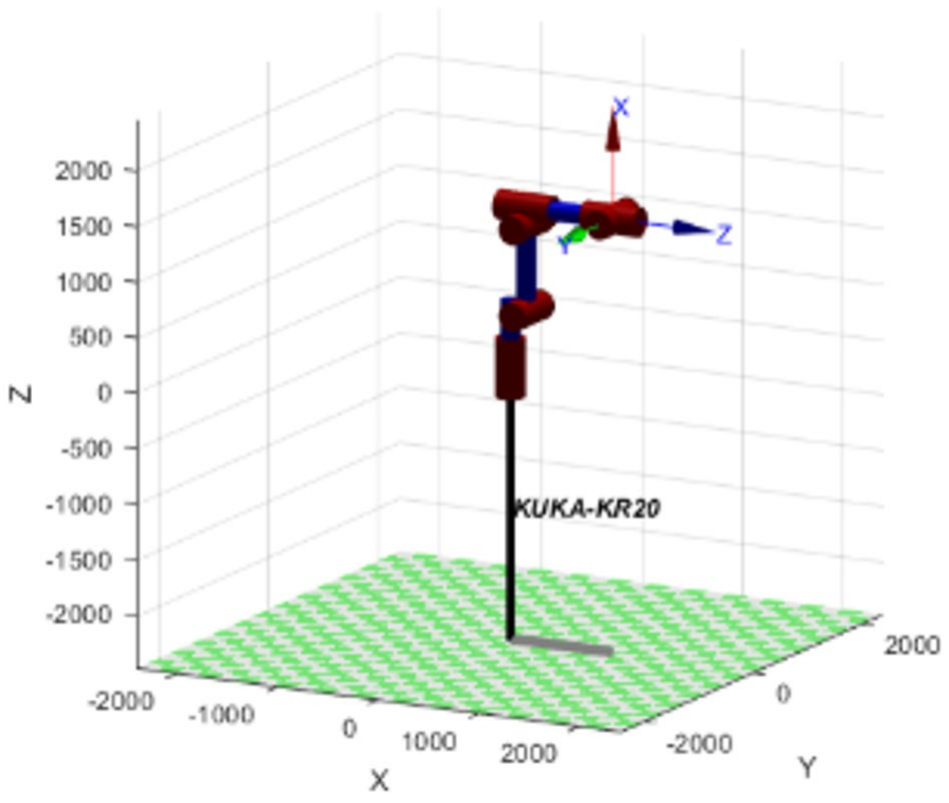
Robotic arm model.

In trajectory planning for a 6-DOF robotic manipulator, the end-effector must transition smoothly through a defined sequence of spatial waypoints without subjecting the mechanical joints to sudden jerks. To navigate through five distinct points (Start-point, three Via-points, and Goal-point) while ensuring the robot starts and stops smoothly, a single continuous polynomial is employed in joint space. The representation of KUKA KR20-R1810 using MATLAB robotics Toolbox as described in Table 13. Is shown in Fig. 11.

Once the discrete waypoints of the end-effector are defined in Cartesian space, inverse kinematics is applied to map these spatial coordinates into their corresponding joint-space angles. Subsequently, polynomial interpolation is utilized across these discrete angular configurations to construct a continuous, time-parameterized motion function for each joint. Since we have 9 specific boundary conditions, an 8th -order polynomial is used as shown in Eq. (21).

21 $$\:{\theta\:}_{j}\left(t\right)={a}_{0}+{a}_{1}t+{a}_{2}{t}^{2}+{a}_{3}{t}^{3}+{a}_{4}{t}^{4}+{a}_{5}{t}^{5}+{a}_{6}{t}^{6}+{a}_{7}{t}^{7}+{a}_{8}{t}^{8}$$

Where $\:j=\mathrm{1,2},\:\dots\:,6$ denotes the joint number, the coefficients *\:a* of the polynomial can be found jointly by Eq. (22), Eq. (23), and Eq. (24). It is known that for the start point $\:{S}_{j}$, intermediate path points $\:{V}_{\mathrm{j}1}$, $\:{V}_{\mathrm{j}2}$, $\:{V}_{\mathrm{j}3}$, and end point $\:{G}_{j}$ of joint *\:j*, the positions, velocities, and accelerations between the five points are continuous. Both velocity and acceleration at the start and end points start at *\:0* and end at *\:0*. As a result, the matrix M can be obtained as shown in Eq. (22). The $\:{t}_{1}$, $\:{t}_{2}$, $\:{t}_{3}$, and $\:{t}_{4}$ in Eq. (22) denote the 4-segments interpolation time of joint *\:j*, respectively. Equation (23) represents the known joint angles and boundary limits.

22 $$\:M=\left[\begin{array}{ccccccccc}1&\:0&\:0&\:0&\:0&\:0&\:0&\:0&\:0\\\:0&\:1&\:0&\:0&\:0&\:0&\:0&\:0&\:0\\\:0&\:0&\:2&\:0&\:0&\:0&\:0&\:0&\:0\\\:1&\:{t}_{1}&\:{t}_{1}^{2}&\:{t}_{1}^{3}&\:{t}_{1}^{4}&\:{t}_{1}^{5}&\:{t}_{1}^{6}&\:{t}_{1}^{7}&\:{t}_{1}^{8}\\\:1&\:{t}_{2}&\:{t}_{2}^{2}&\:{t}_{2}^{3}&\:{t}_{2}^{4}&\:{t}_{2}^{5}&\:{t}_{2}^{6}&\:{t}_{2}^{7}&\:{t}_{2}^{8}\\\:1&\:{t}_{3}&\:{t}_{3}^{2}&\:{t}_{3}^{3}&\:{t}_{3}^{4}&\:{t}_{3}^{5}&\:{t}_{3}^{6}&\:{t}_{3}^{7}&\:{t}_{3}^{8}\\\:1&\:{t}_{4}&\:{t}_{4}^{2}&\:{t}_{4}^{3}&\:{t}_{4}^{4}&\:{t}_{4}^{5}&\:{t}_{4}^{6}&\:{t}_{4}^{7}&\:{t}_{4}^{8}\\\:0&\:1&\:2{t}_{4}&\:3{t}_{4}^{2}&\:4{t}_{4}^{3}&\:5{t}_{4}^{4}&\:6{t}_{4}^{5}&\:7{t}_{4}^{6}&\:8{t}_{4}^{7}\\\:0&\:0&\:2&\:6{t}_{4}&\:12{t}_{4}^{2}&\:20{t}_{4}^{3}&\:30{t}_{4}^{4}&\:42{t}_{4}^{5}&\:56{t}_{4}^{6}\end{array}\right]$$

23 $$\:\theta\:={\left[\begin{array}{ccccccccc}{S}_{j}&\:0&\:0&\:{V}_{\mathrm{j}1}&\:{V}_{\mathrm{j}2}&\:{V}_{\mathrm{j}3}&\:{G}_{j}&\:0&\:0\end{array}\right]}^{T}$$

24 $$\:a={M}^{-1}.\theta\:={\left[\begin{array}{ccccccccc}{a}_{0}&\:{a}_{2}&\:{a}_{2}&\:{a}_{3}&\:{a}_{4}&\:{a}_{5}&\:{a}_{6}&\:{a}_{7}&\:{a}_{8}\end{array}\right]}^{T}$$

### Simulation and trajectory planning results

This section evaluates the performance of the POA against five competitive algorithms in addressing the time-optimal trajectory planning problem. The trajectory is defined by five discrete waypoints, with the corresponding Cartesian coordinates and their derived joint-space angles as summarized in Table 14. For all evaluated metaheuristics, the population size was initialized at 50, the maximum number of iterations was restricted to 100, and the decision variables vector was defined with a dimensionality of four variable $\:{t}_{1}$, $\:{t}_{2}$, $\:{t}_{3}$, and $\:{t}_{4}$. To validate the motion profiles, all simulations were performed using the Robotics System Toolbox in MATLAB.

**Table 14:** Path points in Cartesian and joint space.

Path points	*\:X*	*\:Y*	*\:Z*	$\:{\theta\:}_{1}$	$\:{\theta\:}_{2}$	$\:{\theta\:}_{3}$	$\:{\theta\:}_{4}$	$\:{\theta\:}_{5}$	$\:{\theta\:}_{6}$
*\:S*	1800	0	670	0	0	90	0	0	0
$\:{V}_{1}$	1111.25	1111.25	1221.54	45	45	45	35	15	150
$\:{V}_{2}$	0	1571.54	1221.54	90	45	45	0	25	180
$\:{V}_{3}$	-1111.25	1111.25	1221.54	135	45	45	-35	35	90
*\:G*	-1800	0	670	180	0	90	-45	45	90

**Fig. 12:**
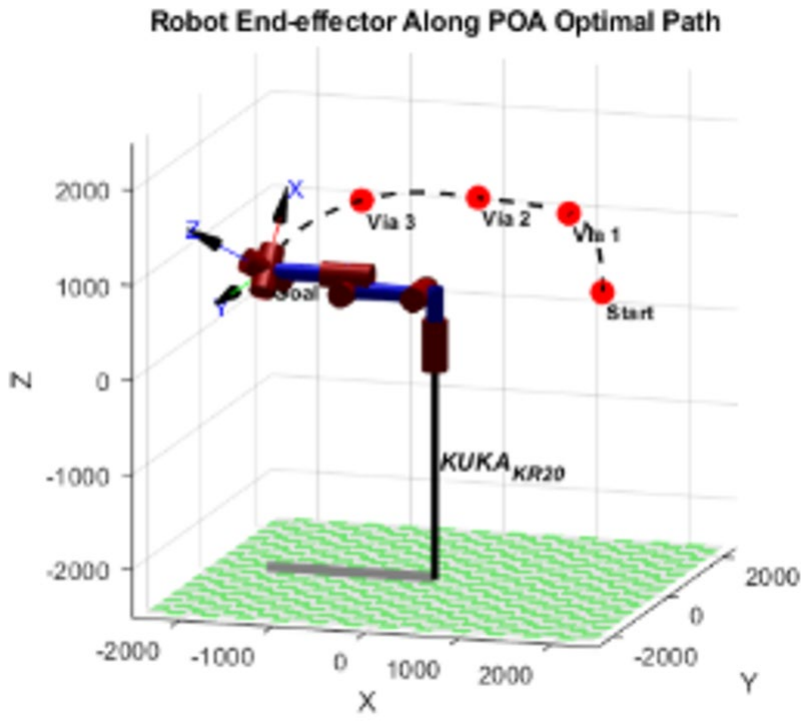
Mechanical arm trajectory operation diagram.

The time-optimal trajectory planning outcomes are detailed in Table 15, as achieved by the proposed POA and five state-of-the-art algorithms: PSO, WOA, HHO, Moth-Flame Optimization (MFO)^34^, and Circle Search Algorithm (CSA)^35^. and the mechanical arm trajectory that obtained by POA is shown in Fig. 12.

**Table 15:** Trajectory planning results for POA and its competitors.

Algorithm	Run time	$\:{t}_{1}$	$\:{t}_{2}$	$\:{t}_{3}$	$\:{t}_{4}$	$\:{t}_{total}$
POA	2.4692	0.8127	0.4344	0.5314	0.5932	**2.3717**
HHO	5.8197	0.8876	0.5198	0.4513	0.6874	2.5461
PSO	2.7287	0.8153	0.4325	0.5266	0.6004	2.3748
WOA	2.3995	0.8976	0.3931	0.4232	0.8159	2.5298
CSA	2.4654	0.8188	0.4205	0.5181	0.6162	2.3736
MFO	3.6891	0.8219	0.4271	0.5202	0.6028	2.3719

**Fig. 13:**
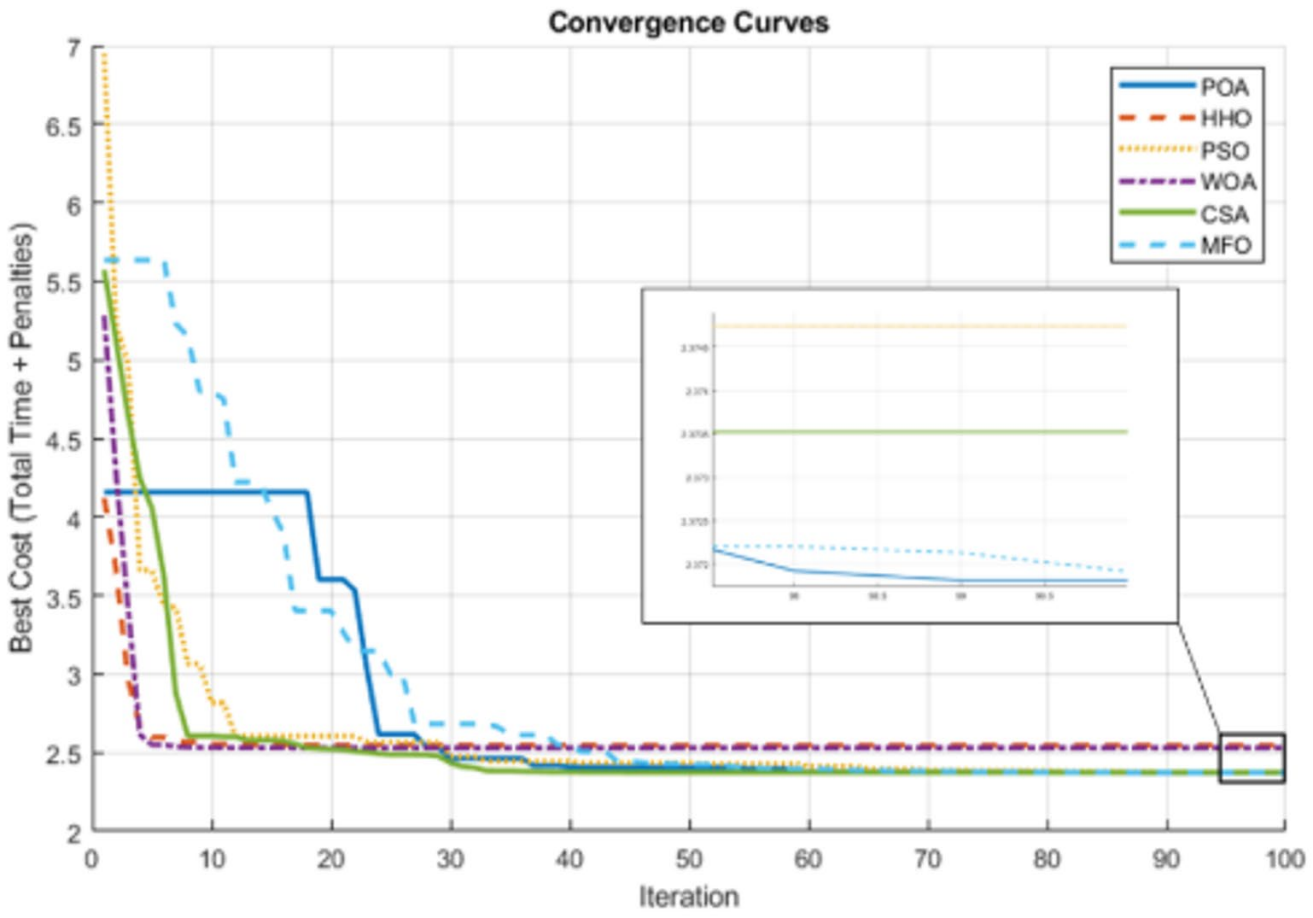
Convergence curves of the different algorithms on trajectory planning.

The results detailed in Table 15 demonstrate that the POA successfully achieved the most optimal trajectory execution time among all evaluated metaheuristics. The POA secured the lowest total trajectory time outperforming all competitive algorithms. This indicates that the POA is highly effective at navigating the search space to find the most time-optimal kinematic path. While algorithms like MFO, CSA, and PSO achieved total times very close to the POA (all within the 2.37 range), the POA still maintained the absolute minimum value, proving its precision in fine-tuning the final motion profile. Overall, the POA outperforms the second-best algorithm, MFO, by a margin of $\:0.01\%$, and demonstrates a $\:0.13\%$ improvement over PSO. Furthermore, it achieves a significant $\:6.85\%$ reduction in total execution time compared to HHO, which yielded the lowest performance in this comparison. the convergence curves presented in Fig. 13 demonstrate the POA’s high convergence accuracy during the trajectory planning process.

**Fig. 14:**
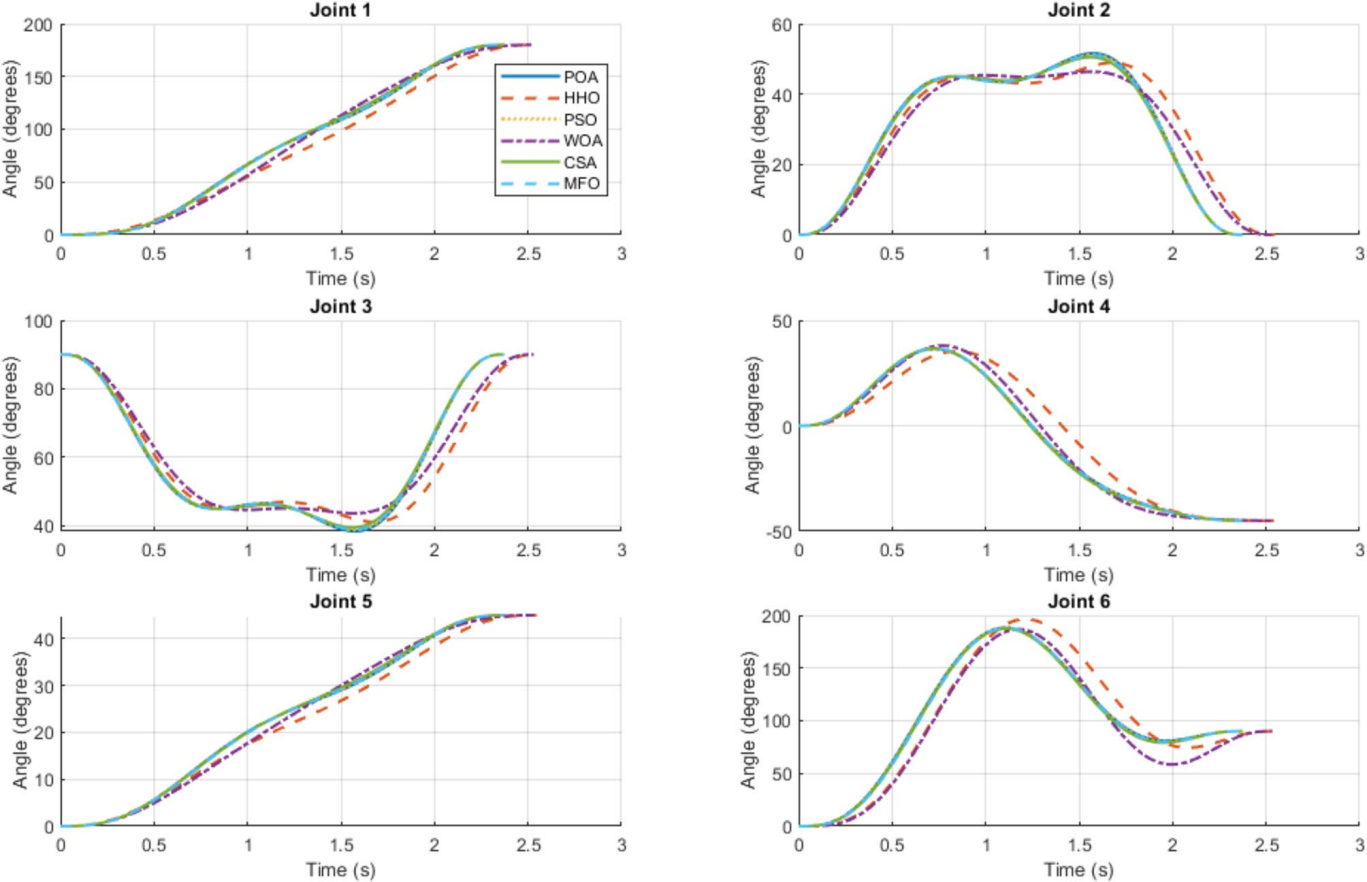
Joints position curves.

**Fig. 15:**
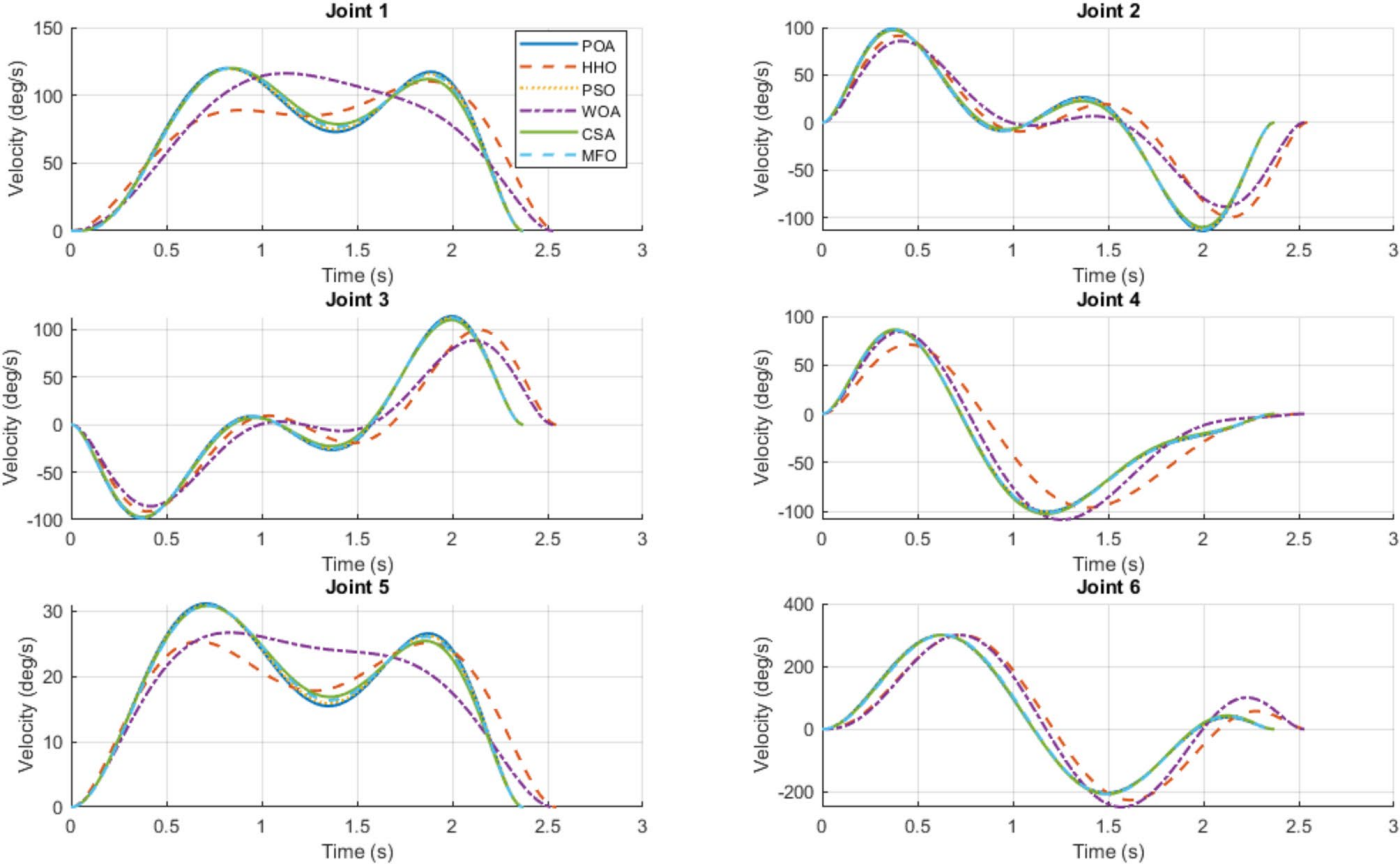
Joints velocity curves.

**Fig. 16:**
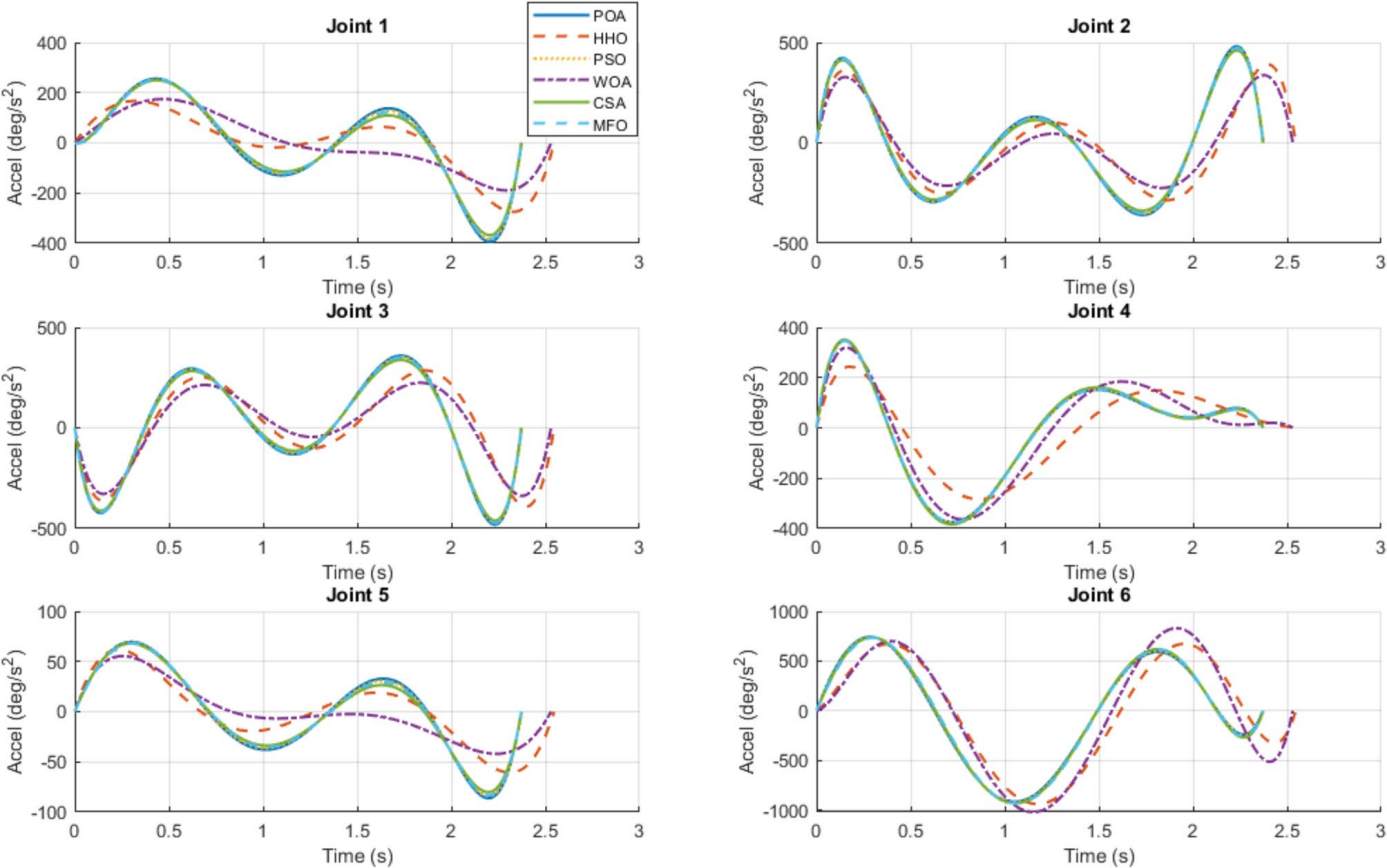
Joints acceleration curves.

The position, velocity, and acceleration profiles of the joints are shown in Figs. 14 and 15, and Fig. 16, respectively. As illustrated in these figures, the generated trajectory for each joint of the robotic arm is kinematically smooth, exhibiting continuous angular velocity and angular acceleration profiles without abrupt fluctuations. The maximum recorded angular velocity reaches $\:300^\circ\:/s$ at joint *\:6*, and the initial and final velocities for all joints correctly rest at zero. These outcomes successfully satisfy the established kinematic constraints, thereby validating the practical feasibility of the proposed algorithm.

## Conclusions and future works

In this paper, we introduce a novel human-inspired, swarm-based metaheuristic algorithm termed the Pray Optimization Algorithm (POA), which is modeled after the procedural rituals of Islamic congregational pray. The algorithm operates through three distinct phases: moving to the mosque, lining up for pray, and the cumulative score phase. During the initial ‘moving to the mosque’ phase, search agents freely navigate the environment to locate a suitable mosque, facilitating broad exploration of the search space in early iterations before transitioning to targeted exploitation of the global optimum. In the second phase, candidate solutions undergo a structured alignment process, mimicking congregants lining up in rows behind the Imam. Finally, in the ‘cumulative score’ phase, each candidate attempts to maximize its respective score, driving the swarm toward the global best solution and significantly accelerating algorithmic convergence.

The performance of the POA was rigorously evaluated on the CEC2017 benchmark suite, proving its efficacy in complex optimization scenarios. Comparative analyses against six established algorithms (PSO, WOA, HHO, COA, RFO, and AOA) confirmed the superior optimization precision of the POA. These performance gains were proven statistically significant via the Wilcoxon rank-sum test, and the Friedman test ranked the POA as the leading algorithm overall. Beyond theoretical benchmarks, the practical applicability of the POA was validated through its deployment on three real-world engineering design problems.

Furthermore, in the domain of industrial robotics, the proposed algorithm demonstrated superior capabilities in the time-optimal trajectory planning of a 6-DOF robotic manipulator. When compared to five state-of-the-art methods (PSO, WOA, HHO, MFO, and CSA), the POA successfully outperformed its competitors, achieving the most efficient execution. Ultimately, the comprehensive results confirm that the POA provides a robust, high-performance solution for theoretical function optimization, structural engineering design, and advanced robotic control. Based on the experimental results above, we have identified several evident strengths and limitations of POA:

- Statistical evaluations indicate that the proposed POA exhibits robust optimization capabilities across both 50- and 100-dimensional CEC2017 benchmark functions. Furthermore, convergence analyses of the 100-dimensional problems illustrate the algorithm’s capacity to effectively escape local optima while refining exploitation precision throughout the iterative process. Scalability assessments confirm that the POA maintains a high degree of optimization accuracy regardless of dimensional expansion. Finally, nonparametric statistical validations _specifically the Wilcoxon rank-sum and Friedman tests_ conclusively verify the significance and reliability of the algorithm’s performance.
- Regarding its limitations, the proposed POA does not uniformly outperform all comparative algorithms across the entire CEC2017 benchmark suite; however, it maintains robust performance margins closely aligned with leading competitors. A structural limitation also exists in its problem-domain applicability: the POA is explicitly designed for continuous, single-objective optimization. As such, applying the algorithm to binary or multi-objective tasks falls outside its current capabilities.

Future research will focus on enhancing the POA by integrating advanced mathematical mechanisms, such as opposition-based learning and chaotic mapping, to further improve optimization efficiency. Additionally, developing a multi-objective variant of the algorithm represents a primary goal for future iterations. Finally, the application of the POA will expand beyond time-optimal trajectory planning to address other critical challenges in robotics, including the minimization of energy consumption and mechanical vibrations.

## Data Availability

The datasets used and/or analyzed during the current study available from the corresponding author on reasonable request.
